# Current Knowledge on the Background, Pathophysiology and Treatment of Levodopa-Induced Dyskinesia—Literature Review

**DOI:** 10.3390/jcm10194377

**Published:** 2021-09-25

**Authors:** Michał Hutny, Jagoda Hofman, Aleksandra Klimkowicz-Mrowiec, Agnieszka Gorzkowska

**Affiliations:** 1 Students’ Scientific Society, Department of Neurorehabilitation, Faculty of Medical Sciences in Katowice, Medical University of Silesia, 40-752 Katowice, Poland; jagoda.hofman98@gmail.com; 2 Department of Internal Medicine and Gerontology, Faculty of Medicine, Medical College, Jagiellonian University, 30-688 Kraków, Poland; Aleksandra.Klimkowicz@mp.pl; 3 Department of Neurorehabilitation, Faculty of Medical Sciences, School of Medicine, Medical University of Silesia, 40-752 Katowice, Poland; agorzkowska@sum.edu.pl

**Keywords:** Parkinson’s disease, abnormal involuntary movements, dopaminergic signalling, basal ganglia, spiny projection neurons, neurotransmission, deep brain stimulation

## Abstract

Levodopa remains the primary drug for controlling motor symptoms in Parkinson’s disease through the whole course, but over time, complications develop in the form of dyskinesias, which gradually become more frequent and severe. These abnormal, involuntary, hyperkinetic movements are mainly characteristic of the ON phase and are triggered by excess exogenous levodopa. They may also occur during the OFF phase, or in both phases. Over the past 10 years, the issue of levodopa-induced dyskinesia has been the subject of research into both the substrate of this pathology and potential remedial strategies. The purpose of the present study was to review the results of recent research on the background and treatment of dyskinesia. To this end, databases were reviewed using a search strategy that included both relevant keywords related to the topic and appropriate filters to limit results to English language literature published since 2010. Based on the selected papers, the current state of knowledge on the morphological, functional, genetic and clinical features of levodopa-induced dyskinesia, as well as pharmacological, genetic treatment and other therapies such as deep brain stimulation, are described.

## 1. Introduction

Treatment of Parkinson’s disease (PD) is inseparable from the use of levodopa, l-3,4-dihydroxyphenylalanine (L-DOPA). Physiologically, it is a precursor molecule in the synthesis of catecholamines that act as neurotransmitters in nervous system—epinephrine, norepinephrine (NE) and dopamine (DA). Decreased DA levels, associated with a degenerative process in the substantia nigra (SN), are the trigger for the cardinal motor features of PD, such as bradykinesia and rigidity [1]. The first symptoms of PD appear when DA levels in the striatum are reduced by 80%, with a corresponding neuronal DA loss of 60% [2]. During this period, treatment is in “the honeymoon phase” [1]. L-DOPA administration alleviates motor symptoms of PD, such as bradykinesia and rigidity, without the presence of abnormal involuntary movements (AIMs) [1]. As the disease progresses, DA biosynthesis and storage decreases. When the dose of L-DOPA entering the brain across the blood–brain barrier exceeds the dopamine storage capacity of dopaminergic neurons, there is an increased dopaminergic activity, leading to hyperkinetic movements—levodopa-induced dyskinesias (LIDs) [1]. They occur at the peak dose, with an increase or decrease in the drug concentration, or both, which is referred to as biphasic LID. As the duration of L-DOPA therapy increases, the level of DA required to induce LID decreases [3].

Motor complications in PD patients develop after varying lengths of therapy. The prevalence of LIDs varies between the sources [4,5,6]; however, about 30% of PD patients develop dyskinesia in the first 5 to 6 years of L-DOPA treatment [5,6]. This fact naturally raises the question, what are the determinants of their occurrence and severity? Another important question is whether there are any methods by which PD patients with LID can be helped to improve their quality of life. This article attempts to answer these questions by reviewing the literature published on this topic in the last decade.

## 2. Materials and Methods

Database searches (PubMed, PubMed Central, Medline) were conducted over a 4-month period, from November 2020 to February 2021. The search strategy was based on creating input keywords consisting of “levodopa-induced dyskinesia” and the following related terms: “genetic”, “therapeutic”, “background”, “pathogenesis”, “therapy”, “treatment”, “deep brain stimulation”, “dopamine receptor”, “dopaminergic activity” and “signal”. The filters applied to the results limited them to English language papers published since 2010. Reviews, systematic reviews, personal communication, letters to the editor, conference material and case reports were not included in the study. The online Mendelian Inheritance in Men database was used as a reference for gene loci and terminology.

## 3. Results

The findings were divided into sections on background (structural and functional; genetic; clinical) and treatment, divided into preclinical studies (Section 3.5) and clinical trials (Section 3.6). The criteria were the purpose of the studies—studies describing the involvement of neuroreceptors, genetic and clinical factors in the development of dyskinesias and the corresponding anatomical and functional changes in brain tissue were classified in the background section, while studies focusing on measuring the efficacy and safety of therapeutic strategies in controlling dyskinesias were classified in the treatment section.

It must be noted, however, that the studies presented below were performed on different subjects. Dyskinesia in animal models may be very different from dyskinesia in humans, which limits the translation of the results in animal studies to practice in humans. The animal subjects that resemble human PD and the LID phenotype the most are primates lesioned with methyl-4-phenyl-1,2,3,6-tetrahydropyridine (MPTP). They present symptoms typical for human PD—rigidity, postural tremor, akinesia—which can be controlled using L-DOPA treatment [7,8]. MPTP primates also experience non-motor symptoms of PD, such as cognitive deficits and sleep disturbances [9]. A limitation of this study model is that the subjects may develop a different severity of parkinsonism (mild vs. advanced) depending on the response to MPTP lesioning [8]. Another issue, which concerns not only MTPT primates, but also 6-hydroxydopamine (6-OHDA)-lesioned rodent models, is the fact that lesioning is an acute induction of pathology in animal, which contrasts with the progressive and chronic nature of the neurodegenerative pathology that is PD [10]. The other most popular animal models are mentioned before 6-OHDA lesioned rodent, including rats and mice. Due to obvious differences in morphology between humans and rodents, dyskinetic movements in the latter case must be measured using different tests. Dyskinesia in a rodent is usually measured with the AIMs scale, measuring forelimb (hyperkinetic movements of contralateral forelimb), axial (twisted posture), orolingual (empty jaw movements) and locomotive (circular movements) dyskinesia [11]. L-Dopa treatment in these animals is reflected by the development of dyskinesia [12]. The topography of lesioning in a PD model rodent is also an important issue, as the medial forebrain bundle lesioning results in creating the subjects with severe and evenly distributed DA denervation, whereas lesioning of the striatum leads to a heterogeneous distribution of DA denervation [13]. Bearing all the above in mind, the readers are advised to use Table S1, (see Supplementary Materials: “Table S1: Summary of reviewed articles concerning the studied subjects and their parkinsonian/dyskinetic phenotypes.”), which clearly describes each enrolled study concerning the type of studied subjects and their “parkinsonian” and “dyskinetic” phenotype.

### 3.1. Structural and Functional Background

#### 3.1.1. Grey and White Matter

From the very beginning of PD, magnetic resonance imaging (MRI) shows anatomical changes in the brain. These involve white matter hyperintensities (WMHs), and grey matter changes in the size of striatum. Both of these phenomena were examined for the significance of their intensity to the development of LID.

In animal model studies comparing severely dyskinetic 6-OHDA-lesioned rats with saline treated rats, it was shown that the striatum size as well as the asymmetry between the sides increased with the increasing duration of L-DOPA treatment, with a predominance on the lesioned side. The degree of asymmetry between sites in the size of the dorsal striatum components, putamen and caudate nucleus, at the onset of PD, was found to be a predictor of the development of LID, but also of the response to L-DOPA treatment [3]. The microscopic changes, although evident, did not appear to be directly related to striatal growth or the axial, limb and orolingual AIMs scores, measured together using established criteria of amplitude and severity [14].

The primary motor cortex (M1) in animals has also been shown to undergo changes associated with PD progression. In the rats that developed LID, an increase in the dendritic spike density was observed that was significantly correlated with the severity of LID. The dendrites of tested neurons mainly received glutamatergic excitatory signals; therefore, the increased density of dendritic spines was thought to reflect the development of hypersensitivity to glutamatergic input in these neurons. These results suggest a role for M1 neurons in the development of LID [15]. In humans, although M1 volume was significantly negatively correlated with the development and severity of diphasic LID, no such relationship could be proven for density; therefore, only volume can be considered as a potential marker of diphasic dyskinesia in patients [16].

Lesioning the striatum of rats decreases the dopaminergic innervation in M1 ipsilateral to the lesion. It is represented by the loss of tyrosine hydroxylase fibres, whose density in the lesioned side is 75% lower than in the side contralateral to the lesion. Lesioning also influences the levels of M1 monoamines and their metabolites. The dopamine and norepinephrine levels are decreased, but administration of L-DOPA restores the level of DA back to the regular values [17].

A retrospective analysis of 484 patients showed an association between WMHs and LID in patients with PD. The results showed that periventricular, lobar, basal ganglia and infratentorial hyperintensities were associated with the occurrence of dyskinesia. Although such a result provides evidence of an association between lesions and LID, it also indicates that any WMHs’ location is not dominant in influencing the development of LID [18].

#### 3.1.2. Basal Ganglia

Neurons of basal ganglia: the internal globus pallidus (GPi) and subthalamic nucleus (STN) present an altered firing rate in dyskinetic patients. Both grouped discharges, as well as low frequency firing, are significantly more frequently found in this group [19]. In MPTP parkinsonian non-human primates, chronic treatment with L-DOPA alters the activity of basal ganglia without changing the striatal DA release [20]. It also increases interactions between the STN and the entopeduncular nucleus, which, in rodents, is a structure analogous to human GPi [21]. Therefore, it is possible that changes in the firing pattern of basal neurons contribute to the development of LID [19,20]. Although the electrophysiological parameters of STN are not associated with the degree of severity of AIMs, the attenuation of rodent STN rebalances the activity of D1R and D2R signalling pathways, which results in the decreased severity of LID [22].

#### 3.1.3. Cerebellum

Cerebellar circuits are specifically addressed in studies of LID development. The functional connectivity (FC) of the cerebellum and other parts of the brain at rest was studied in groups of PD patients without LID, PD patients with LID and controls. The functional connectivity between the motor cerebellum and posterior cortical and cerebellum in PD patients is higher than in non-PD patients. The presence of LIDs is positively correlated with an increase in the FC between lobule VIIIb and the left inferior frontal gyrus [23]. Cerebellar involvement in dyskinesia is also associated with red nucleus (RN) iron levels. This parameter is elevated in the PD patients with dyskinesia, whereas it remains unchanged in the PD patients without dyskinesias. As the RN is involved in cerebellar circuits, it is thought that the iron level in RN reflects an increased activity of cerebellar pathways. Their increased activity may be a compensating mechanism in response to the development of LID [24].

#### 3.1.4. Circulation and Angiogenesis

Local cerebral blood flow (CBF) is increased under hypercapnia in areas of vascular proliferation. D1R activity stimulates vasodilatation and angiogenesis [25]. Abnormally elevated levels of the putaminal cerebral glucose metabolism rate (CMR) are observed during an OFF phase in both dyskinetic and non-dyskinetic patients. These high CMR values decrease after the administration of L-DOPA. It also causes an increase in putaminal CBF values in 75% of dyskinetic PD patients and in 50% of non-dyskinetic PD patients. The difference between the two groups of patients, although it exists, is not significant enough; therefore, neither CBF nor CMR can be considered as an indicator of changes leading to the development of LID in patients with PD. On the other hand, the parameter that significantly differentiates dyskinetic and non-dyskinetic patients is the resting activity of the primary somatosensory cortex and M1. In addition, the topography of dyskinetic symptoms reflects cortical areas that show abnormal CBF and CMR baseline values [26]. Although there is no significant difference in the CBF and CMR values, these parameters can be further used to determine the putaminal hyper-perfusion/hypometabolism index (PHI). It compares the ON-phase putaminal perfusion and glucose metabolism with values measured for the thalamus, which is a structure similar in size to the putamen that is not affected by D1R activity. The PHI reflects abnormal activity of the putamen after the administration of L-DOPA. The presence of LID in PD patients is associated with higher PHI values. It is possible that this parameter can be used as a marker to assess the risk of developing LID in patients with PD [27].

#### 3.1.5. Alterations in Cerebrospinal Fluid

Cerebrospinal fluid (CSF) analysis is one of the basic neurological examinations providing a very useful insight into the CNS state. Significant differences in the DA and NA turnover rate in CSF are observed between dyskinetic and non-dyskinetic PD patients (higher in the dyskinetic group). In PD patients not treated with L-DOPA, a decrease in the CSF concentrations of the following metabolites: dihydroxyphenylacetic acid (DOPAC) and homovanillic acid, is observed. Physiologically, DA concentrations increase as the age-dependent DOPAC/DA ratio decreases. These observations suggest DA in CSF, as well as the concentrations of their metabolites, are potential prognostic markers for the development of LID [28], although they need confirmation in future studies.

#### 3.1.6. Metabolic Changes

A DA deficiency results in an impaired metabolism of neurotransmitters—mainly glutamate (Glu), glutamine and aspartate in the following specific brain regions: midbrain and right cortex. Parkinsonian and dyskinetic rats show lower levels of synaptophysin compared to control animals, which may indicate reduced synaptic plasticity. Synaptophysin is a glycoprotein of presynaptic vesicle membranes, responsible for the docking and fusion of neurotransmitter-carrying vesicles. Its levels were shown to decrease with age and to be reduced in neurological disorders such as PD and Alzheimer’s disease [29]. Lower levels of synaptophysin and postsynaptic density protein 95 (PDS95) are correlated with reduced dendritic spine density [30], which impairs synaptic plasticity [31]. Changes in astrocyte activity, resulting from elevated myo-inositol levels, may also contribute to the development of dyskinesia [32].

### 3.2. Genetic Background

The latest technologies offered by genetics and molecular science have been used to study the genetic determinants of dyskinesias in Parkinson’s disease. Single nucleotide polymorphism (SNP) in the *GALNT14* (2p23.1; MIM 608225) gene (rs144125291) [33], as well as in the gene for the GRIN2A subunit of the NMDA receptor (rs7192557 and rs8057394) [34] are significantly correlated with LID [33]. *GALNT14* encodes information concerning one of the Golgi proteins involved in glycosylation. It may, therefore, be involved in the basal levels of neuroinflammation in the brain [33]. The *MAOB* gene polymorphism (MIM 309860)—rs1799836, G allele—is associated with an increased risk of LID development, when compared to the A allele [35]. The levels of long non-coding RNA (lncRNA) molecules, such as NONRATT023402, are altered in dyskinetic rats. The regulatory targets of this lncRNA molecule are the *Gsto2* (10q25.1; MIM 612314) and *Ptger3* (1p31.1; MIM 176806) genes, which are associated with PD and LID [36].

As the DA levels decrease during PD, gene expression is altered in the spiny projection neurons (SPNs) of the direct (dSPNs) and indirect (iSPNs) pathways. These changes are particularly evident in the genes regulating MAPK signalling [37]. The difference between dSPNs and iSPNs in the level of expression of Ap-1, ERK and cyclic AMP-responsive element binding protein-dependent genes is clear, with more changes occurring in dSPNs. Cellular homeostatic mechanisms counteract these changes, but without an effect on Ap-1-dependent gene expression [37]. The genes associated with the development of AIMs—*Sstr2* (17q25.1; MIM 182452), *Sstr4* (20p11.21; MIM 182454) and *Kcnn3* (1q21.3; MIM 602983)—are overexpressed in dSPNs [37].

Chronic L-DOPA treatment leads to changes in the DNA methylation activity of parkinsonian rats by increasing the expression levels of genes for DNA demethylases, *Tet3* (2p13.1; MIM 613555) and *Gadd45b* (19p13.3; MIM 604948). These epigenetic changes correlate with the downregulation of other genes’ expression, as a result of prolonged L-DOPA administration. They are also associated with an increased expression of genes involved in synaptic plasticity [38]. Bromodomain and extraterminal family proteins bind to acetylated histones, thus playing an effector role in transcriptional regulation. Their dysfunction has been associated with the reorganisation of dorsal striatal chromatin and with changes in corticostriatal plasticity [39].

### 3.3. Alterations of Neurotransmission

A dopamine transporter (DAT) is a presynaptic membrane molecule responsible for transporting DA to neuronal cells. The lower activity of DAT membrane expression is thought to reflect the depletion of dopaminergic neurons [40]. The expression level can be measured using single-photon emission computed tomography, where the quantity of DATs is reflected by the specific binding ratio (SBR) of a radiotracer [41]. Using the same technique, it is possible to examine changes to the pre- and post-synaptic membranes of dopaminergic neurons. It was shown that the uptake of a tracer for the DAT decreased significantly more in PD patients than in healthy controls. The condition of the post-synaptic membrane, evaluated using a radiotracer for D2R and D3R, did not differ between the PD patients and the healthy controls [42]. Measurement of the putaminal SBRs of the radiotracer for the DAT in a 4-year follow-up study showed a significant reduction in signal in the PD patients who developed dyskinesia [43].

Using a functional MRI technique, it is possible to investigate which connections between the cortex and striatum are modulated by chronic L-DOPA treatment and evaluate the effect of the dopaminergic modulation of connections on the severity of LID in patients with established dyskinesia, as well as in non-dyskinetic PD patients [44]. An acute dose of L-DOPA triggers changes in dopaminergic modulation (via feedback connections from the putamen to the M1 and presupplementary area (preSMA)), although the extent of the provoked response differed significantly between the patients with and without LID. Increased signalling between the putamen and M1 is thought to account for the inadequate ‘Go’ response observed in NoGo trials [45]. No-Go activity in the preSMA has also been shown to be a predictive measure for the severity of dyskinesia. Given the overactivity of putamen in LID, this structure may be a key factor in the development of dyskinesia [46].

Dyskinesia can be induced without administration of L-DOPA—by the reactivation of striatal neurons. As an animal study shows, inhibition of their activity thus leads to a lack of dyskinetic response to L-DOPA. Neurons in striatum are mainly SPNs, but the γ-aminobutyric acid (GABA)-ergic fast spiking interneurons (FSIs) and iSPNs are also present in this brain region [47]. The activity of dSPNs decreases with dopamine depletion in the midbrain, resulting in disproportion in the activity of dSPNs and iSPNs [48,49]. After the administration of L-DOPA, the activity of dSPNs increases again, while the activity of iSPNs decreases, leading to a restoration of balance [48,49]. In LID, the effect of L-DOPA on the activity of both SPN populations is enhanced [48,49]. The activation of dSPNs alone is sufficient to induce LID, as demonstrated by laser light provocation. Both selective D1R and D2R agonists activate a comparable number of striatal neurons, and both induce LID, although selective D1 agonists trigger more vigorous changes in the glow rate and more severe dyskinetic symptoms [48]. Interestingly, the antiparkinsonian effect of L-DOPA is also mediated to a greater extent by D1R than D2R [50]. The activity patterns of SPNs are altered in the PD and LIDs of MPTP parkinsonian primates. Dopamine depletion is associated with an increased burst spike frequency or pause loss. Due to the complexity of interactions between dopaminergic and glutamatergic systems, the exact mechanism leading to these changes remains unclear. As mentioned earlier, dopaminergic signalling provokes a response that is different for the SNP subpopulations, resulting in an imbalance of their activity [51].

The striatum and cortex of a parkinsonian brain are bidirectionally connected in rats [52]. As the disease enters an advanced stage, these connections change their characteristics [53]. It is unclear whether the connections become unidirectional [54] or whether the observed imbalances are due to the increased strength of one direction relative to the other [52]. Dyskinetic events are associated with a frequency peak of 80 Hz, in both an animal model and humans [52,54,55]. Long-term potentiation (LTP) and long-term depression (LTD) are mechanisms that contribute to synaptic plasticity. Long-term potentiation occurs in indirect pathways in parkinsonian mice and in direct pathways during dyskinesias, whereas LTD occurs in direct pathways in parkinsonian mice and in indirect pathways during dyskinesias [54]. Thus, dyskinesia is not triggered by impaired LTP induction, but results from a reduced excitability difference between dSPNs and iSPNs during the ON-phase [54,55,56].

#### 3.3.1. Inhibitors of Monoamine Oxidase B and Catechol-O-Methyltransferase

Both of these enzymes are one of the key components of DA metabolism. Their inhibitors are widely used as adjuvant therapy in the treatment of PD with L-DOPA [57]. Identification of the SNPs of genes for catechol-O-methyltransferase [58] and monoamine oxidase B (MAO-B) [59] was thought to be useful for estimating the activity of these enzymes, which, in turn, influences DA metabolism and, indirectly, dyskinetic symptoms [58,59].

#### 3.3.2. Dopaminergic Pathways and Dopamine Receptors

The results of the studies on rodent parkinsonian model animals showed that three distinct signalling pathways—PKA/DARPP-32, ERK and mTORC1—play an important role in the mechanism of LID development [60,61]. They are all activated by a common intracellular cascade that is triggered in striatonigral SPNs expressing D1Rs [60,61]. Long-term L-DOPA administration increases the activity in these pathways, leading to changes in striatal bidirectional synaptic plasticity [61]. The activation of one pathway may affect the activity of the other pathways [61]. DARPP-32 signalling modulates the ERK and mTORC1 pathways [60,61]

Recent studies have closely examined the effect of D1R activation on ERK1/2 through interaction with the tyrosine phosphatase Shp-2 [62]. Not only has Shp-2 activation been shown to be a key factor for ERK1/2 and mTOR activation via phosphorylation and, consequently, for LID expression, but it has also been demonstrated that long-term stimulation with intermittent L-DOPA or D1R agonists leads to Shp-2 phosphorylation. This effect can be counteracted by D1R antagonists, consequently influencing the levels of the D1R/Shp-2 pathway end products—p-mTOR and p-ERK1/2 [62]. Rapamycin was shown to selectively affect mTORC1 in the striatum without affecting either the PKA/DARPP-32 and ERK pathways or the expression of the α-amino-3-hydroxy-5-methyl-4-isoxazolepropionic acid receptor (AMPA) and NMDA receptor subunits [61].

In lesioned animal models, the potential of D1R to associate with PKA and ERK1/2 was increased, and this change occurred concomitantly with the onset of LID [63]. However, within 2 weeks of treatment with L-DOPA, D1R was not able to activate the PKA and ERK1/2 pathways. These observations indicate a remodelling of the direct striatal pathways that results from the oversensitivity of dSPNs [63]. The rate of D1R-dependent ERK1/2 phosphorylation is modulated by mGluR5, which alters the mGluR5/PLC/PKC pathway through the modulation of calcium/dependent responses. However, it does not influence the PKA activity [64]. The hypersensitivity of the D1R/cAMP/PKA pathway is associated with the elevation of the Gα_olf_ protein level. This protein is the major striatal stimulating G-protein, whose activity leads to increased cAMP levels. Interestingly, despite the decreased Gα_olf_ protein levels in mutant animals, the D1R/cAMP/PKA pathway was found to be hypersensitive in these subjects. Therefore, the activity of Gα_olf_ may not be essential for the hypersensitivity of this pathway [65]. Chronic L-DOPA administration is associated with increased levels of Gα_olf_ protein in striatonigral SPNs of parkinsonian mice, and a decreased level of this protein in striatopallidal SPNs [66].

Knocking-out casein kinase 2 (CK2) is also associated with reduced G_olf_ levels in striatonigral SPNs [67]. The knockout (KO) of CK2 in striatonigral neurons in model animals results in a decreased severity of dyskinesia. This also correlates with lower pERK levels in dSPNs and cholinergic aspiny interneurons (ChIs). When CK2 is knocked-out in striatopallidal neurons, the severity of dyskinesia and the pERK levels in dSPNs and ChIs increase. However, in the latter case, the LID can be attenuated by coadministration of caffeine with L-DOPA [67].

An increased expression of Nurr1 transcription factor in striatal neurons of dyskinetic rats is associated with the increased cortically evoked firing rate and the increased spine density of SNPs [68]. Both downstream D1R/D2R activation pathways, PKA and ERK1/2, are influenced by the activity of leucine-rich repeat kinase 2 (LRRK2). The interactions between these pathways and LRRK2 are not yet well understood, but their further investigation may result in a new therapeutic strategy to modulate the downstream activity of dopaminergic receptors, leading to better control of LID [69]. The dopaminergic system in the midbrain is also regulated by the activity of vesicular glutamate transporter type 3 in a circadian-dependant matter [70].

The surface distribution of dopaminergic receptors is dictated by anchoring protein-PSD95 [71]. Its levels are increased in dopamine-depleted brains, causing lower diffusion of D1R in the membrane. The association between DA loss and increased levels of PSD95 might be involved in the development of dyskinesia in non-human parkinsonian primates [72]. The activity of parkin, an E3 ubiquitin-protein ligase, is impaired not only in genetic parkinsonism, but also in sporadic PD patients. In animal models, the loss of function of this enzyme leads to the sequestration of ubiquitinated proteins. It is associated with D1R-mediated decreased proteasome catalytic activity, which is reflected by the severity of AIMs [73].

Ca^2+^/calmodulin-dependent protein kinase IIα (CaMKIIα) binds to the intracellular domain of D2R. The Gαi domain of D2R is also localised in this region [74]. This may indicate that CaMKIIα affects D2R activity in the adenylyl cyclase signalling pathway. In parkinsonian rats, the level of interaction between CaMKIIα and D2R is elevated. Decreasing its level leads to the attenuation of LID with an efficacy similar to D2R agonists [75].

An increased D3R response is observed in animals with severe dyskinesia, which is associated with an increased GABA release. This differs significantly from animals with lower AIMs scores. D3R can affect the ERK pathway independently of D1R, thus adding up to the effect of D1R modulation [76]. The deletion of D3R is associated with decreased levels of FosB, ERK and H3 activity. It also causes an attenuation of dyskinesia, with preservation of the therapeutic effect of L-DOPA [77]. The main locus of D3R, whose levels are elevated in dyskinetic patients, is the GP [78]. A lack of D5 signalling in D5R animals is associated with the decreased therapeutic effect of L-DOPA and the increased expression of LID. As LID increases, LTD plasticity is reduced in these animals. Decreased acetylcholine (ACh) release also negatively affects the activation of M1 postsynaptic receptors in dSPNs [79].

#### 3.3.3. GTPases of Ras Family

Raf/Mek/ERK is a pathway that activates the ERK signalling cascade. It is stimulated by Ras GTPases, whose activity is increased by DA and glutamate receptors. Decreasing the striatal levels of Ras-guanine nucleotide-releasing factor (Ras-GFR1) leads to inhibition of the Ras/ERK pathway in parkinsonian mice. Ras-GFR1 affects the Ras-ERK pathway only in dSPNs, without affecting the cholinergic interneurons of ChIs [80]. It, therefore, affects specific downstream D1R pathways, consequently inhibiting the development of hypersensitivity to DA stimulation in the neurons. This is associated with LID developing less, although dopaminergic stimulation retains its therapeutic effect on parkinsonian motor symptoms [80,81].

The administration of drugs that modify the MEK-ERK core components further potentiates the aforementioned antidyskinetic effect [82]. Eliminating Ras-GRF1 decreases ERK, PKA and mTOR signalling intensity [83]. Consequently, the Ras-GRF1 levels in striatonigral SPNs also affect LTP-dependent synaptic plasticity. Such a relationship was not observed in striatopallidal SPNs, which may be due to the involvement of DARPP-32 or Ras-GRF2 activity [84]. The Ras-ERK pathway in mice can be overactive due to the overexpression of Ras-GRF1, or due to the reduced gene expression of the Ras inhibitor (Nf+/−). Interestingly, in both cases, the severity of dyskinesia does not exceed normal levels. However, the administration of lovastatin, a Ras inhibitor, improves the control of dyskinetic symptoms only in the group with normal Nf+/− gene expression [85].

#### 3.3.4. FosB Transcription Factor

Chronic treatment with L-DOPA leads to an increased level of the truncated splice variant of FosB (ΔFosB). Among others, this transcription factor regulates the genes responsible for plasticity in striatal neurons. Elevated levels of ΔFosB are associated with a loss of FosB function, which results in the early development of dyskinesia and unstable firing rate changes during the ON phase in L-DOPA-treated animals [86]. MSH1 histone kinase activity also increases with ΔFosB levels, consistent with MSK1′s target, histone H3. H3 is localised on c-fos-associated nucleosomes, and its phosphorylation results in an increased expression of this gene [81]. In SPNs expressing D1R, MSK1 also decreases the Polycomb-group-proteins-dependent gene repression in parkinsonian mice [87].

The level of c-Fos expression in M1 neurons differs between sides in an animal model of unilateral MFB injury after acute (*p* = 0.003) or chronic (*p* = 0.001) L-DOPA treatment, although it is elevated when compared to the control. It is also positively correlated with the total AIMs score (*p* = 0.13). Another immediate-early gene product, ARC, which is associated with synaptic plasticity, showed no such correlation [17]. Both of these genes are overexpressed in the dorsolateral bed nucleus of the terminal stria (dlBST) in dyskinetic rats. This structure is not included in the basal ganglia, and as such, has not been implicated in the development of LID. Interestingly, inhibition of ∆FosB expression in dlBST results in a reduced AIMs score [88]. The neuronal NO synthase inhibitor 7-nitroindazole also downregulates FosB/ΔFosB expression and reduces dyskinetic symptoms in a rodent model [89].

#### 3.3.5. Glutamatergic Signalling

Neurotransmission systems, other than those that are dopaminergic, also affect SPNs and contribute to the development and expression of LID. One of these is the glutamatergic system. As the duration of L-DOPA therapy increases, NMDAR activity and its composition changes [90]. Interactions of Rph3A protein with the GluN2A subunit of NMDARs are increased in parkinsonian dyskinetic rats. Altering the PSD95 function leads to improved AIMs scores in animals. This may be a promising target for translation to humans as an antidyskinetic therapy, despite the potential post-synaptic interactions involving PSD95 [91]. This protein also contributes to the overactivity of NMDAR in dyskinetic animals. It modulates interactions between Fyn kinase and the NR2B subunit [92]. AMPA antagonists directly acting on the glutamate receptors in M1 are effective in reducing the severity of dyskinesia. NMDA antagonists did not alter dyskinesia in parkinsonian rats, although simultaneous action on NMDA and AMPA receptors increased the effect of AMPA antagonism [93].

#### 3.3.6. Cholinergic Signalling

The density of nicotine cholinergic receptors (nAChRs) in the striatum of dyskinetic PD patients remains normal, in contrast to the reduced levels of nAChRs in non-dyskinetic patients [94]. This indicates that cholinergic activity is preserved in LID, which may be a mechanism of overcoming DA loss in striatal neurons. Cholinergic activity induces DA release from otherwise serotoninergic neurons. The regulatory mechanisms of dopaminergic transmission do not affect the above DA release, as evidenced by the lack of significant differences in the measurement of DAT binding in the striatum between dyskinetic and non-dyskinetic patients [94].

The metabotropic muscarinic cholinergic receptors (mAChRs) of PD dyskinetic rats, specifically M1R and M4R, are involved in LID expression. Interestingly, M4R has a bidirectional function—endogenous ACh inhibits LID and striato-nigral SPN activation via M4R stimulation, whereas during LID, it facilitates its effect on postsynaptic M4 mAChRs. The exogenous stimulation of mAChRs in M4, presumably at presynaptic dopaminergic terminals or ChIs, also potentiates an inhibitory effect on LID [95]. The activation of M4Rs by endogenous ACh potentiates LTD and inhibits LTP in the dSPN glutamatergic synapses of animal models. Thus, the function of M4Rs is similar to that of D2R in iSPN. The exogenous stimulation of M4R increases synaptic plasticity in dSPNs and thereby attenuates LID [96].

#### 3.3.7. Serotonergic Signalling

Serotonergic neurons are capable of converting 5-HT into DA, storing the produced DA, and then releasing it. This function is referred to as the presynaptic serotonergic LID mechanism [97,98]. Surgical or pharmacological lysis of serotonin raphe projections in dyskinetic rats has been shown to block LID, providing evidence for a key role of dopamine released from serotonergic neurons in the pathogenesis of LID [99]. The observed greater severity of dyskinesia in animals with a large number of serotonergic endings in the striatum is not due to DA release per se but rather to an abnormal activation pattern (with a predominance of D1R over D2R) and lack of an adequate DA gradient [100]. A study in rodent and primate models of PD, as well as post-mortem studies of brain tissue examination, have shown significant upregulation of the 5-HT transporter (SERT) in the striatum of subjects experiencing LID. The expression of dyskinesia was correlated with a radiotracer binding to SERT [97]. Brain neurotrophic factor (BDNF) overexpression, which can be caused by L-DOPA treatment, has been shown to correlate with axonal growth of serotonin neurons in the striatum and putamen of dyskinetic rats, which was further associated with the increased activity of the cAMP/PKA and mTORC pathways [101]. In the DA-depleted striata of PD model monkeys, SERT+ axons were significantly denser in the sensorimotor part of the striatum. The synaptic incidence was also significantly increased in the area of dopaminergic denervation [102]. The above results suggest that synaptic plasticity and proliferation of serotonergic neurons, which follow the dopaminergic denervation and further L-DOPA treatment, may be important factors in the development of motor symptoms in PD, as well as in LID [97,101,102]. In dyskinetic rats, selective serotonin reuptake inhibitors (SSRIs) such as citalopram, paroxetine and fluoxetine show a dose-dependent antidyskinetic effect when administered together with L-DOPA. In contrast, no such effect was observed in animals treated with apomorphine, a direct DA agonist. This indicates a presynaptic mechanism of antidyskinetic action [103,104]. L-DOPA administration does not affect 5-HT receptor activity, although it reduces the effect of SSRI on the neuron firing rate in the dorsal raphe nucleus (DRN) [105].

### 3.4. Clinical and Environmental Background

Certain non-modifiable factors and clinical features are associated with an earlier onset or higher severity of LID. Female gender is an independent risk factor for developing LID. Patients whose tremor predominates over rigidity and akinesia have been shown to have a lower risk of LID [3], but these data are inconclusive. In another study, patients who initially had a tremor had a significantly higher risk of LID during follow-up [106]. The course of treatment also influences the characteristics of LID in PD. A higher mean dose of L-DOPA during the first 6 months of therapy [6], as well as greater severity of motor symptoms [107], are significantly associated with an early onset of LID. These observations are mutually consistent—patients who initially have more severe parkinsonian motor symptoms require higher doses of L-DOPA to restore their normal motor function [3]. Moreover, the need for more rapid escalation of the drug dose is a direct reflection of the rapid degeneration of the dopaminergic system and correlates with an earlier onset of LID [106]. It has been pointed out, however, that it is not clear whether L-DOPA administration is a contributing factor to the development of LID itself, or rather just a trigger [3]. Nonetheless, a less-pulsatile administration of L-DOPA, e.g., 6× doses per day, has been shown in clinical trials to reduce the risk of LID development in both PD patients who have never previously initiated L-DOPA treatment and in non-dyskinetic patients treated with L-DOPA [108]. A continuous supply of L-DOPA via an enteral levodopa-carbidopa gel reduces fluctuations in DA levels. In patients with dyskinetic symptoms lasting more than 4 h/day, a reduction in LID duration, severity and accompanying pain was observed after the administration of a levodopa-carbidopa enteral gel. In patients with a duration of dyskinetic episodes less than 4 h/day, the duration of dyskinesias increased, albeit with a significant reduction in severity and pain [109].

Adenosine A2A receptor antagonists may reduce the severity of dyskinesia, which is associated with greater receptor availability in PD patients who develop LID [110]. Caffein, as an A2A antagonist, may be a factor delaying the onset of LID [111]. The N-of-1 study on nicotine use demonstrated a significant effect of nicotine on the occurrence of LID compared to a placebo [112].

### 3.5. Treatment-Preclinical Studies

#### 3.5.1. Modification of L-DOPA Delivery and Release

As LIDs are motor symptoms caused by pulsatile changes in DA concentration due to intermittent L-dopa administration, the obvious strategy to prevent them is the continuous delivery and release of the drug (continuous dopamine stimulation; CDS)—this way, theoretically, the DA concentration will reach a stable level at which the therapeutic, antiparkinsonian function is active without LID side effects.

L-DOPA/benserazide poly(lactic-glucosic acid) (LBM) microspheres are a novel therapeutic option for the treatment of PD. In animals, LBM showed a dose-dependent antiparkinsonian effect and did not significantly increase AIMs scores. In control animals treated with L-DOPA, the D1R/Shp-2/ERK1/2 pathway activity increased with duration and onset of dyskinesia, which was not observed in LBM-treated animals [113]. These results are consistent with a previous study that also demonstrated the efficacy of LBM therapy in reducing LID’s frequency [114]. Abnormal PKA/DARPP-32 signalling leads to the increased phosphorylation of tau protein, which in turn activates SPNs and results in LID. PKA/DARPP-32 and tau protein phosphorylation activity was increased in the mice with parkinsonism treated with standard i.p. L-DOPA/benserazide (LS) therapy. A dose-dependent inhibition of PKA/DARPP-32 and tau protein phosphorylation was observed in the LBM-treated animals [115].

Striatal PKA-related glutamatergic signalling and glutamatergic receptors (AMPA) may play an important role in the expression and occurrence of LIDs. During chronic repeated L-DOPA treatment, the AMPA receptor subunit, GluR1, becomes over-phosphorylated at the serine-831 and serine-845 position. Such changes (elevated levels of pGluR1S831 and pGluR1S845) are not found in LBM-treated animals [116].

An alternative method of L-DOPA delivery is chitosan-coated nanoliposomes (CCN). ERK1/2, DARPP-32 and FosB/ΔFosB expression and LID induction are significantly less elevated in parkinsonian rats treated with CCN, compared to the same animal model treated with LS. However, these parameters in PD rats treated with CCN are higher than in a control group of saline-treated animals [117].

#### 3.5.2. Amantadine Counteracts LID

Amantadine, an antiparkinsonian drug, is used to reduce LID. In a study conducted in an animal model, the administration of amantadine decreased the severity of LID episodes. Interestingly, GABA release in SN, which was increased after L-DOPA treatment and during AIMs, increased significantly less when L-DOPA was administered concomitantly with amantadine. This suggested that the mechanism of the antidyskinetic effect of amantadine may be related to an effect on nigral GABA levels [118]. Recent studies have shown that amantadine inhibits dyskinesia by blocking the inward-rectifying type 2 K^+^ channel in SPNs rather than NMDA receptors [57].

#### 3.5.3. Serotonin in the Treatment of LID

The serotoninergic system is largely present in the basal ganglia. Chronic L-DOPA treatment and a decreased DA concentration leads to its alterations [119]. It is speculated that drugs that modify 5-HT activity may have an inhibitory effect on LID. However, they require careful administration because an overdose of serotonin agonists leads to 5-HT syndrome, the symptomatology of which is similar (tremor and rigidity) to PD [120]. The 5-HT1A partial agonist buspirone inhibits LID in rats by influencing the 5-HT1A receptors, although it does not alter STN electrical activity [121]. It also regulates GABA and Glu release and burst activity in pars reticular of SN (SNr) [122].

The 5-HT1A agonist 8-hydroxy-2-dipropylaminotetralin (8-OH-DPAT) significantly reduces LID, but this benefit is outweighed by the 5-HT syndrome that is caused by 8-OH-DPAT [123]. Nonetheless, 8-OH-DPAT delays LID development [123] and reduces elevated cortical gamma activity that was caused by D1 and D2 agonism [124]. The efficacy of SSRI (citalopram) in inhibiting dyskinesia in rats is similar to 8-OH-DPAT and buspirone, but it does not induce significant 5-HT syndrome [125]. LID inhibition by another 5-HT1A agonist, BMY-14802, increases adequately with the dose (20 > 10 > 5 mg/kg). The antidyskinetic effect of BMY-14802 was observed not only in LID induced by L-DOPA administration, but also in LID induced by the administration of D1 and D2 agonists. However, this effect can be reversed by using 5-HT1A receptor antagonists in L-DOPA-induced LID [126].

The 5-HT3 antagonism was another serotoninergic system checkpoint that was investigated for its potential to reduce LID. In rats, the first significant differences in the AIMs scores were observed after 9 days of treatment with ondansetron (5-HT3 antagonist) and L-DOPA, compared with animals treated with L-DOPA alone. This difference steadily increases. Interestingly, ondansetron administration was discontinued on day 23, resulting in significant differences between the L-DOPA and ondansetron and the control groups on day 30. These results suggest the potential of 5-HT3 antagonists, such as ondansetron, in controlling LID in the treatment of PD, although further research is necessary in order to understand this correlation [127].

The serotonin precursor molecule, 5-hydroxy-tryptophan (5-HTP), has also been studied to control LID. The use of 5-HTP increases the cytoplasmic levels of serotonin, which, by competing with DA in serotonergic neurons, decreases the amount of DA released by these neurons [128,129]. Dyskinetic rats with 6-OHDA damage were administered different doses of 5-HTP, L-DOPA, L-DOPA and 5-HTP or L-DOPA and 5-HTP and a 5-HT antagonist. After evaluating the motor functions of the test animals, 5-HTP was shown to have antidyskinetic potential. It also inhibited the development of dyskinesias in parkinsonian rats treated from the beginning with the combination of L-DOPA and 5-HTP. The control of parkinsonism symptoms by L-DOPA was not worsened when 5-HTP was used [128].

Finasteride, a 5-α-reductase inhibitor, is involved in neurosteroid synthesis [130]. This is a group of molecules involved in the physiology of nervous tissue and the pathophysiology of its diseases, including PD [131]. Neurosteroids interact with the dorsal raphe nucleus and influence serotonergic activity [132], also by regulating the DA release from serotonergic neurons [130]. Since the latter is an important factor in the development of LID, finasteride has been studied in dyskinetic rats for its antidyskinetic potential. The results of this study suggest that it may be effective in controlling LID in both acute and chronic treatment, alone or in combination with L-DOPA [130].

#### 3.5.4. β-Adrenoceptor Blockade—Propranolol

To date, the modulation of LID by adrenergic compounds has emphasised the α-adrenergic receptor (αAR), despite the high density of β-adrenergic receptors (βAR) in the striatum of PD patients [133]. The optical isomer (−) of propranolol affects βARs in the dorsal striatum and is the isomer responsible for reducing LID, with a two-fold longer antidyskinetic effect compared with (±) compounds [134]. Although propranolol alone can reduce the motor activity of rats, which is a highly undesirable effect in the treatment of PD, there is a broad doses spectrum in which this reduction does not occur, with a concomitant significant effect on LID. Supportive therapy with propranolol normalises aberrant signalling in the striatum of rats [134]. Propranolol could not attenuate LID induced by D1 and D2 receptors agonists, indicating that propranolol acts through a presynaptic mechanism [135]. Due to the association between the reduction in striatal DA, propranolol administration and the reduction in LID reduction, it was suggested that the antidyskinetic mechanism of propranolol may be related to the reduction in striatal DA levels. It may be caused by the secondary anticholinergic effect of propranolol. An inhibition of βARs results in a decrease in intracellular cAMP, which leads to a decrease in the spontaneous firing rate of ChIs, resulting in a decrease in ACh levels. Striatal DA release is regulated by ACh, and therefore, the effect of propranolol on ACh levels could possibly restore the balance between ACh and DA in LID, which should be examined in the further studies [136].

#### 3.5.5. Nicotinic Receptor and Its Agonists

The acetylcholine receptors (AChR) of nigrostratial neurons modulate neurotransmission, including dopaminergic signalling [137]. Nicotine, a direct agonist of nAChR, has been shown to significantly reduce AIMs scores after 8 (*p* < 0.01) and 12 (*p* < 0.05) weeks of administration in rats. Although acute nicotine administration stimulates nAChR, thereby increasing dopamine release [138], intermittent nicotine treatment desensitises nAChR. It also decreases dopamine release, which may explain its antidyskinetic effect—in later stages of PD, DAT levels are reduced, resulting in a less efficient clearance of excess DA [139]. Interestingly, a more recent experiment showed no significant improvement in dyskinesia in female mice after a 10-week treatment of nicotine, as well as in the group treated for 5 days with α7 nAChR partial agonist (product name: AZD0328). Brain-derived neurotrophic factor levels in the striatum, but not in the prefrontal cortex, were shown to be significantly correlated with the development of LID. Nicotine failed to significantly reduce high BDNF levels in the striatum but succeeded at the prefrontal cortex level. It was suggested that the reason for the lack of modulation of LID by the drugs tested is due to the condition of the animals—their nigrostriatal damage was almost complete, indicating that the treatment of LID with nicotine requires a less damaged nigrostriatal pathway [140].

#### 3.5.6. Ionotropic Receptors of Glu

The previously described increase in glutamatergic transmission in the development of PD and LID indicates that its inhibition appears to be an attractive therapeutic approach for the treatment of LID. As the disease progresses, an increase in the NMDA subunits, NMDA-R1 and NMDA-R2, acting as an ion channel and regulatory unit, respectively, becomes apparent [90]. Studies on Compound Formula Rehmannia showed its inhibitory effect on NMDA-R1 and NMDA-R2 expression. It also increased the expression of GABA-RB1, which becomes down-regulated during LID. This effect was correlated with the decrease in LID in test animals [141]. Memantine, a non-competitive NMDAR antagonist, exhibits a similar mechanism through which it attenuates LID in rat models of PD [142].

#### 3.5.7. Histamine H_2_ Receptor Antagonism

Given the widespread distribution of H_2_ receptors in the basal ganglia involved in the development of LID, both in their input and output regions [143], it is conceivable that H_2_ receptors may be targets for antidyskinetic therapy. Chronic treatment with ranitidine reduces LID expression in 6-OHDA rats treated with L-DOPA. A suggested mechanism of action of ranitidine to reduce dyskinesias involves the inhibition of the PKA pathway in the SPNs [144]. Ranitidine inhibits striatal Glu efflux, as well as GABA release in SNr, thus affecting the pre- and postsynaptic mechanisms of LID development. The pre-treatment of rats with ranitidine reduces AIMs scores, without reducing the efficacy of anti-PD treatment [145].

#### 3.5.8. Opioid Modulators

As opioids affect neurotransmission in the basal ganglia and the intensity of opioid signalling is significantly altered during PD [146], drugs that modulate this transmission system appear to be an effective alternative to standard antidyskinetic therapy. Treatment with a combination of L-DOPA and nalbuphine (κ receptor agonist and μ receptor antagonist) reduce the expression of ΔFosB in the striatum of MPTP-lesioned parkinsonian primates, as well as other LID markers, such as prodynorphin, dynorphin A, Cdk5, Thr34-phosphorylation of DARPP-32, to normal values. Drug administration did not cause any AEs, and even the sedative effect present in nalbuphine monotherapy was not induced by L-DOPA administration [147]. The significant results of this experiment may be due to the dualistic nature of the treatment strategy used—the highly selective µ-opioid receptor antagonism did not improve dyskinesia in the tested 6-OHDA rats [148]. Only μ receptor agonists appeared to reduce the incidence of LID in MPTP parkinsonian primates when used alone [149].

Nociceptin/orphanin FQ opioid peptide (NOP) receptors, despite some functional similarities, differ from opioid receptors—they are not activated by the most standard opioid receptor ligands. Although both NOP agonists, AT-390 and AT-403, exhibited dose-dependent overall hypolocomotor effects in parkinsonian rats, AT-403 produced a significant, albeit mild, improvement in dyskinesia [150].

#### 3.5.9. cAMP and cGMP Signalling–Inhibitors of Phosphodiesterase 10A

Phosphodiesterase 10A (PDE10A) is an enzyme expressed exclusively in brain tissue, including the SPNs expressing D1R and D2R [151]. Hydrolysing cyclic nucleotides cAMP and cGMP affects DA signalling in SPNs, showing an effect similar to D2R antagonists. In PD patients, PDE10A expression is significantly reduced in the striatum and GP. Due to the presence of PDE10A in both populations of SNPs, it is possible that its theoretical antidyskinetic effect could exceed that shown by D2R antagonists [152]. In MPTP parkinsonian primates, a novel PDE10A inhibitor (product name: MR1916) showed high penetration into the brain and antidyskinetic effects greater than amantadine over the entire dose range—not only with acute but also with chronic administration. It also did not cause significant AEs [153].

#### 3.5.10. Genetic Treatment

Another therapeutic approach to LID focuses on modifying the expression of LID-related genes. Considering the latest techniques used in molecular sciences, this area appears to be a very promising and effective strategy, and genes related to the dopaminergic system and dopaminergic pathways seem to be the first choice. In parkinsonian rats, interesting results were obtained by the overexpression of DA autoreceptor D2Rs in the DRN, which is involved in the previously described uncontrolled uptake and release of L-DOPA in serotonergic neurons. The DRN, in which D2Rs were overexpressed, was insensitive to high, LID-inducing doses of L-DOPA and dopaminergic agonists. This demonstrated that genetic control of serotonergic DRN neurons could be used to reduce dyskinesia in studied animals [154]. Inhibition of the p11 gene (1q21.3; MIM 114085) in the dorsal striatum of parkinsonian mice produced surprising results. Its product, S100A10, is known to activate the serotonin 5-HT1B receptor, which, in turn, is associated with decreased LID. Thus, downregulating p11 expression should rather increase LID, and, in fact, it inhibited dyskinesia more effectively than the direct pharmacological activation of 5-HT1B. These results suggested that the effect of p11 on LID development may function through mechanisms not involving 5-HT1B [155]. The transplantation of dopaminergic neurons has also been investigated for the effect of L-DOPA on graft survival. Both allogeneic and xenogeneic transplants were viable but showed no significant improvement of motor functions in the test animals. Furthermore, post-transplantation L-DOPA treatment induced an immune response in xenogeneic grafts [156]. It has been shown that ERK hypersensitivity, secondary to striatal damage in rats, can be reduced by overexpression of G protein-coupled receptor kinase (GRK) (two-fold). This also resulted in decreased Akt activity and decreased ΔFosB levels in the striatum. Increasing GRK expression with lentivirus may be a potential strategy to reduce dyskinesia, although this strategy requires further examination [157]. A direct target of GRK, a G protein-coupled receptor, is also β-arrestin2, a protein scaffolding various intracellular molecules [158]. In rats with β-arrestin2 overexpression, a significant reduction in LID efficiency for L-DOPA was observed, without affecting the therapeutic efficacy of L-DOPA. Knocking out β-arrestin2 had the opposite effect of increasing dyskinesia [159,160]. Knocking out adenylyl cyclase 5 (AC5) appeared to be another option to modulate downstream dopaminergic pathways. A significant attenuation of LID was observed in AC5 KO mice, coupled with decreased activation of the PKA pathway, resulting in a decreased ERK level, as well as lower FosB/ΔFosB expression [161].

Knocking out the α5 subunit of nAChR in 6-OHDA-lesioned mice leads to the attenuation of dyskinesia in these animals. Compared with wild-type individuals, KO mice showed less dopaminergic denervation in SN, as manifested by reduced rotational movements after amphetamine administration. DAT activity was also lower in modified individuals, resulting in less 6-OHDA neurotoxicity [162].

As mentioned earlier, CaMKIIα is involved in LID expression [75]. Modulating its function through inhibitors such as KN-93 has been shown to reduce LID in rats. Although it did not show a significant antiparkinsonian effect, it did reduce pGluR1S845 levels and Gad1 (2q31.1; MIM 605363) and Nur77 (12q13.13; MIM 139139) gene expression, all of which were increased after 3 weeks of L-DOPA treatment [163].

CaV1.3 calcium channels are present in striatal SPNs [164]. The administration of nimodipine (CaV1.3 antagonist) showed a reduction in LID in studies in dyskinetic animals. Recently the effects of CaV1.3 antagonism on LID expression have been studied more directly, by silencing the genes responsible for striatal CaV1.3 channels at the mRNA level. Even partial silencing of CaV1.3 in the striatum led to the complete prevention of LID. The method presented here was designed to specifically target the striatum, as otherwise, silencing CaV1.3 in other tissues could lead to serious cardiac, neurological and psychological consequences [165].

Peroxisome proliferator-activated receptors (PPAR) have been shown to be involved in the antidyskinetic effect of URB597 (an inhibitor of endocannabinoid catabolism) and capsazapine. Moreover, the direct activation of PPARγ led to a decrease in LID in parkinsonian rats, which was also correlated with an increase in ERK phosphorylation [166].

### 3.6. Treatment-Clinical Trials

#### 3.6.1. Modification of L-DOPA Delivery and Release

As mentioned in the clinical background section, one of the CDS methods is the use of levodopa-carbidopa intestinal gel (LCIG) [109]. In patients with advanced PD, the standard of care is a 16-hour long infusion of LCIG, whose superiority over oral L-DOPA in improving the life quality and motor functions in these patients has been repeatedly described in various populations of observational studies and clinical trials (phase three) [167,168,169,170,171,172,173]. It has been shown to reduce dyskinesia by more than 40% [168]. Some recent studies have suggested that, in certain situations, such as sleep disturbances, the 24-hour LCIG infusion may be more beneficial in terms of sleep quality [174] and the improvement of daily time with dyskinesia (phase three) [175]. Previous studies have shown that approximately 30% of patients have experienced serious AEs associated with LCIG therapy [168,172]. Less severe AEs, such as abdominal pain, occurred in up to 87% of patients [168]. Although, in general, LCIG treatment had similar safety levels as standard oral L-DOPA treatment; it also carries a risk of complications associated with gastrointestinal tube insertion guidance [168,169,172,176,177,178]. It was also observed that initiating LCIG monotherapy bears a risk of developing diphasic dyskinesia in patients who have not experienced this type of dyskinesia before. This problem might be resolved by increased morning and continuous LCIG flow or by using ER L-DOPA or DA agonists during bedtime [179]. Despite the presence of the above AEs, LCIG can also be successfully used in older, advanced PD patients [180], although there is a significant risk of therapy discontinuation due to the presence of AEs [98].

Apomorphine was the first DR agonist introduced for the treatment of PD [181]. Continuous subcutaneous apomorphine injection (CSAI) is an apomorphine delivery method that has been used for nearly 40 years in many countries [182] as a monotherapy or adjunct therapy to standard oral L-DOPA therapy. The efficacy of CSAI in controlling dyskinesias varies between studies. It is generally considered a likely option in patients with advanced PD [182,183,184,185,186] and has been described as a transitional option between the standard treatment and DBS, providing better control of dyskinesia [181,187]. However, it has sometimes been pointed out, that the increase in LEDD due to combined treatment with oral L-DOPA and CSAI led to a lack of initial improvement in dyskinesias and was a reason for discontinuing therapy [181,187,188]. In contrast, this problem was not observed with CSAI used in monotherapy, and the positive effect on dyskinesias was beneficial [185].

An ER L-DOPA/carbidopa (CD-LD) (product name: IPX066) prolongs the duration of the ON phase without dyskinesia, shortens the duration of the OFF phase, and does not increase the severity of LIDs in patients studied during phase three clinical trials, compared with the treatment effects of immediate release (IR) CD-LD, and CD-LD with entacapone [189,190]. Concomitant treatment with CD-LD and DA agonists, MAO-B inhibitors, or amantadine does not significantly affect treatment outcomes, although small changes in treatment efficacy have been observed [190].

#### 3.6.2. Amantadine Counteracts LID

Oral immediate-release amantadine is widely used in early PD as an alternative to L-DOPA treatment. In a meta-analysis, the anti-dyskinetic effect of amantadine was proven, also noting its vision-related AEs [191]. It is possible that amantadine may delay and consequently reduce the incidence of LID [192]. Despite its long half-life, it is usually administered in two daily doses, which, due to its high concentrations at night, may lead to sleep disturbances [193].

Extended-release amantadine (ERA) capsules (product name: ADS-5102) significantly (27%; *p* = 0.005) reduce dyskinesia and increase the duration of drug efficacy without dyskinesias in the experimental group compared to the control group (phase three trials) [194,195]. ERA has also been shown to improve MDS-UPDRS (phase two and three trials) [195,196]. The adversary effects (AEs) of long-term ERA therapy were dose-dependent and most commonly included falls, sleep disorders and hallucinations [196]. In most cases, they were not caused by the drug itself, but were due to the severity of the PD course [197]. NMDA-receptor antagonism caused hallucinations, and cholinergic antagonism caused symptoms such as dry mouth, constipation and nausea [194,197,198]

#### 3.6.3. D3 Selective Agonism

Another proposed solution to control LID is to affect D1R activity by modulating D3R activity, either with D3 partial agonists or with D3 antagonists. Both receptors are co-expressed in dSPN and together are able to form a heteromeric D1R-D3R complex [77,199]. Overactivity of D3R, which is observed in dyskinesia [76], increases the membrane binding of D1R, consequently increasing the activity of downstream D1R pathways [200]. Studies in animal models have shown that partial D3 agonists are able to reduce the incidence of LID, which may be due to the restoration of the balance between D1R binding to the membrane and its internalisation [200]. Partial D3 agonists also did not reduce the efficacy of L-DOPA in reducing Parkinsonian symptoms, as observed with D3R antagonists [77,199,201]. Pramipexole is a non-ergoline agonist that prefers D3 receptors. Pramipexol monotherapy (achieved by gradually replacing previously used drugs with pramipexole) led to significant decrease in Core Assessment Program for Surgical Intervention Therapies scores compared with baseline values after 4 weeks of the treatment and was maintained at this level. Patients treated with pramipexole had a significant improvement of MDS-UPDRS part III (improvement in motor symptoms) and quality-of-life after 2 weeks of therapy. The only observed AEs were increased dyskinesia and the prolonged duration of the OFF phase. D3 agonism is a potential candidate for reducing LID in clinical practice because it combines the efficacy of PD treatment, LID control and safety of use. This may be due to the synergic effect of D1 and D3. The agonists preferring D3 may provide stimulation levels to overcome the hypokinetic symptoms of PD, but also low enough to not induce LID [202].

A new drug (product name: IRL790), a selective D3 receptor antagonist, has been proposed [203]. Dyskinetic primates show increased D3R expression in the striatum [201]; therefore, D3 receptor antagonism may be a potential strategy for reducing dyskinesia. D3 receptor antagonists, partial agonists and D3R deletion attenuated the occurrence of LID [77]. During the phase 1b clinical trial, treatment with IRL790 caused a significant number of AEs, with 90.1% of the patients taking D3-selective antagonists experiencing at least one AE, which was less than the AEs in the placebo group. The most common AE in the IRL790 group was worsening parkinsonism. Significantly more AEs were observed during dose titration of the drug than during the dose-size stabilisation phase. Treatment of efficacy was measured using the Unified Dyskinesia Rating Scale, showing an 11.5 point median reduction and an 8.2 point mean reduction in the intervention group compared to the placebo. The drug has been shown to be able to significantly reduce LID, although the concomitant presence of AEs must also be taken into account [203].

#### 3.6.4. MAO-B Inhibition

Safinamide is relatively new drug whose proven mechanism of action is MAO-B inhibition [204]. It has been studied as a potential adjuvant therapy to standard L-DOPA treatment in PD, albeit in a limited population [204,205,206]. The results of these phase III studies did not allow for a firm conclusion on the efficacy of safinamide as an add-on therapy to L-DOPA in patients with advanced PD [204,205,206], neither did studies in a rodent model [207]. AEs associated with safinamide treatment such as cataracts, asthenia, fever, falls, back pain, dyskinesias, worsening of PD, headaches and insomnia have been observed in more than 10% of patients [204,205].

#### 3.6.5. Serotonin in the Treatment of LID

The results of a phase I/IIa trial showed that treatment with 5-HT1A/B agonist, eltoprazine, at a 5.0 and 7.5 mg dose, significantly reduced Clinical Dyskinesia Rating Scale scores, although a significant reduction in the maximum LID severity was observed only at the 5.0 mg dose. No significant changes in MDS-UPDRS-III scale scores were observed. While the above therapeutic results are not as spectacular as those previously reported in animal models, such a difference was to be expected given the differences in the complexity of the substrate of LID in humans and rodents [208].

The antidyskinetic potential of 5-HTP was also studied in a small group of twelve patients with parkinsonism in a phase IIa clinical trial. The efficacy of the drug was measured using UPDRS Part III and IV scores, as well as UDysRS and patient self-report diaries. The results of a 16-week follow-up after treatment with 50 mg of 5-HTP showed significant improvements in all the study scores compared to baseline and the placebo group. Despite the promising results of this study, its limitations must be taken into account, and larger group studies are needed to adequately assess the antidyskinetic potential of 5-HTP in patients with parkinsonism [209].

#### 3.6.6. Histamine H_2_ Receptor Antagonism

Although the results of ranitidine in animal models appear promising, a phase IIa clinical trial with famotidine in a human model showed no significant differences between the treatment effect of famotidine (doses: 80, 120, 160 mg/day) and a placebo. No significant AEs were observed in this study, demonstrating the safety of famotidine. The lack of clinically significant results in this experiment illustrates that the complexity of LID problems in humans cannot always be adequately represented in animal models, and that optimistic results from studies in rats or non-human primates may not necessarily translate to human experiments [210].

#### 3.6.7. Invasive and Non-Invasive Brain Stimulation

Deep brain stimulation (DBS) is an invasive treatment option for advanced PD, in which the therapeutic window is increasingly narrow and pharmacological control of the disease is increasingly difficult. DBS can control both motor symptoms of parkinsonism and dyskinesias [211]. Its primary target is the STN, although the efficacy of stimulation of this area in controlling LID and motor symptoms of PD has recently been compared with the stimulation of GPi [211,212]. Stimulation of the STN is widely used in clinical practice, and as shown in recent studies, is more effective in reducing the equivalent daily dose of L-DOPA (LEDD) compared to GPi-DBS [211,213]. Although the DBS-STN is usually used to treat advanced PD, it was also studied in a phase four clinical trial in PD patients with mild motor complications and a short (1.5 years) history of dyskinesia [214]. The use of DBS-STN resulted in a better treatment outcome. However, AEs were also more frequent than in the pharmacological-only treatment group [214]. Some phase three trials have suggested that GPi stimulation is associated with a more significant reduction in dyskinesia than STN-DBS, but the significance of this difference has not been proven for treatment durations longer than 12 months [212,215]. The results of retrospective analysis showed that STN-DBS improved the MDS-UPDRS IV score in 57–61.9% of patients, depending on the group, with the improvement exceeding the direct effect of LEDD reduction on LID. This effect increased with time after treatment (69% vs. 77% at 1 year vs. 2 years after treatment, respectively) [216]. Interestingly, stimulation of the area above the STN (including the zona incerta) had better efficacy than stimulation of the STN alone (18 vs. 15 patients; *p* = 0.048) [217].

Repetitive preSMA transcranial magnetic stimulation (rTMS) has been studied as an alternative, non-invasive stimulatory intervention meant to control LID [218]. As described previously, preSMA activity is increased in dyskinetic PD patients, which may reflect pathological processes in the development of LID or attempts to compensate the pathology [46]. The results of this study showed that preSMA overactivity is one of the factors contributing to the development of dyskinesia, and that rTMS exhibits an antidyskinetic effect proportional to the strength of the applied magnetic field [218]. This study confirmed the results of previous studies showing the efficacy of rTMS in neurodegenerative diseases, including PD. Low-frequency rTMS has been shown to reduce dyskinesia in PD patients, and high-frequency rTMS has been shown to improve their motor function [219].

## 4. Conclusions

Structural changes in the brain that become apparent as an increase in asymmetry in the striatum size between sides, with a predominance of the degenerate side, leads to the development of LID. Functional changes in the basal ganglia appear to be central in the development of LID, particularly low frequency firing. Bidirectional plasticity in the cortico-striatal pathway is altered. SPNs of the direct and indirect pathway show an altered frequency and pattern of firing in LID. The excitability of dSPNs and iSPNs in the ON-phase is impaired. Dopaminergic downstream pathways—PKA/DARPP-32, ERK and mTORC1—are activated as part of a common cascade, beginning in striatonigral D1Rs SPNs. With the duration of L-DOPA treatment, the activity of these pathways increases. D3R, located mainly in the GP, shows an additive effect on LID, and its deletion reduces the activation of FosB, ERK and H3 histone. Risk factors for the development of dyskinesias include a higher mean L-DOPA dose size over 6 months, a higher dose escalation rate, earlier onset of PD, greater severity of motor symptoms and female gender. New routes of administration of L-DOPA (LBM, CCN) have been introduced, as well as ER compounds LD and CD, and amantadine, with very satisfactory results in lowering LID in advanced PD patients, whereas the standard oral immediate-response compounds of amantadine or apomorphine are useful as adjunct therapy for early (amantadine) or advanced (apomorphine) PD. Selective D3R agonists, such as pramipexole, may be considered as a moderate alternative to L-DOPA. Both 5-HT-1A and 5-HT-1A/B agonists showed dose-dependent antidyskinetic effects. SSRI also reduced LID, without the risk of inducing significant 5-HT syndrome. Propranolol is thought to affect dyskinesia by decreasing the firing rate in ChIs, leading to a decrease in ACh levels and consequently to a decrease in DA release. Although in the clinical practice STN-DBS is the only stimulation applied in the advanced stage of PD, the literature suggests that DBS can be applied to two regions, STN and GPi. Additional stimulation of areas above the STN may have better results than stimulation of the STN alone.

## Supplementary Materials

The following are available online at https://www.mdpi.com/article/10.3390/jcm10194377/s1, Table S1: Summary of reviewed articles concerning the studied subjects and their parkinsonian/dyskinetic phenotypes.
