# Supplementary material for: Current Knowledge on the Background, Pathophysiology and Treatment of Levodopa-Induced Dyskinesia—Literature Review
Source: J Clin Med. 2021 Sep 25;10(19):4377. doi: 10.3390/jcm10194377 (PMC8509231; doi:10.3390/jcm10194377)
Supplement: Supplementary file 1 [file jcm-10-04377-s001.zip › jcm-1347775-supplementary.pdf]

Table S1. Summary of reviewed articles concerning the studied subjects and their parkinsonian/dyskinetic phenotypes.

Ref. no.	Author; year	Subject type	Behaviour in "Parkinson" state	Behaviour in "dyskinetic" state
**[3]**	Eusebi et al.; 2018	PD patients	Resting tremor, rigidity, bradykinesia	Abnormal involuntary movements (twitches, jerks, twisting)
**[6]**	Turcano et al.; 2018	PD patients	Resting tremor, rigidity, bradykinesia	Abnormal involuntary movements (twitches, jerks, twisting)
**[14]**	Fletcher et al.; 2020	6-OHDA MFB-lesioned hemi-parkinsonian rats	Net contraversive rotations	Hyperkinetic movements of contralateral forelimb, twisted posture, vacuous chewing, tongue protrusion
**[15]**	Ueno et al.; 2017	6-OHDA MFB-lesioned hemi-parkinsonian rats	Contralateral rotations after apomorphine injection	Hyperkinetic movements of contralateral forelimb, twisted posture, empty jaw movements
**[16]**	Zhi et al.; 2019	PD patients	Resting tremor, rigidity, bradykinesia	Biphasic dyskinesia (dystonia-improvement-dystonia)
**[17]**	Lindenbach et al.; 2015	6-OHDA MFB-lesioned hemi-parkinsonian rats	Akinesia of the forelimb ipsilateral to the lesion	Hyperkinetic movements of contralateral forelimb, twisted posture, empty jaw movements
**[18]**	Chung et al.; 2020	PD patients	Resting tremor, rigidity, bradykinesia	Abnormal involuntary movements (twitches, jerks, twisting)
**[19]**	Li et al.; 2015	PD patients	Resting tremor, rigidity, bradykinesia	Chorea, choreoathetosis, myoclonus, akathisia, ballism
**[20]**	Porras et al.; 2014	MPTP-lesioned parkinsonian non-human primates	Akinesia, bradykinesia, abnormal posture, absent checking behaviour	Abnormal flowing/flicking movements of limbs; sustained/transient sustained posture of leg, arm, trunk, or neck
**[21]**	Aristieta et al.; 2012	6-OHDA MFB-lesioned hemi-parkinsonian rats	Contralateral rotations after amphetamine sulfate administration	Hyperkinetic movements of contralateral forelimb, twisted posture, empty jaw movements
**[22]**	Aristieta et al.; 2019	6-OHDA MFB-lesioned hemi-parkinsonian rats	Full-body turns ipsilateral to lesion	Hyperkinetic movements of contralateral forelimb, twisted posture, empty jaw movements
**[23]**	Yoo et al.; 2019	PD patients	Resting tremor, rigidity, bradykinesia	Abnormal involuntary movements (twitches, jerks, twisting)
**[24]**	Lewis et al.; 2013	PD patients	Resting tremor, rigidity, bradykinesia	Abnormal involuntary movements (twitches, jerks, twisting)
**[25]**	Jourdain et al.; 2017	PD patients	Resting tremor, rigidity, bradykinesia	Abnormal involuntary movements (twitches, jerks, twisting)
**[26]**	Jourdain et al.; 2016	PD patients	Resting tremor, rigidity, bradykinesia	Abnormal involuntary movements (twitches, jerks, twisting)
**[27]**	Aljuaid et al.; 2019	PD patients	Resting tremor, rigidity, bradykinesia	Abnormal involuntary movements (twitches, jerks, twisting)
**[28]**	Andersen et al.; 2017	PD patients	Resting tremor, rigidity, bradykinesia	Abnormal involuntary movements (twitches, jerks, twisting)
**[32]**	Yang et al.; 2020	6-OHDA MFB-lesioned hemi-parkinsonian rats	Contralateral rotations after apomorphine injection; akinesia of the forelimb ipsilateral to the lesion	Hyperkinetic movements of contralateral forelimb, twisted posture, empty jaw movements
**[33]**	Ryu et al.; 2020	PD patients	Resting tremor, rigidity, bradykinesia	Involuntary choreiform, dystonic body movements
**[34]**	Ivanova et al.; 2012	PD patients	Resting tremor, rigidity, bradykinesia	Abnormal involuntary movements (twitches, jerks, twisting)
**[35]**	Kakinuma et al.; 2020	PD patients	Resting tremor, rigidity, bradykinesia	Abnormal involuntary movements (twitches, jerks, twisting)
**[36]**	Han et al.; 2020	6-OHDA MFB-lesioned hemi-parkinsonian rats	Contralateral rotations after apomorphine injection	Hyperkinetic movements of contralateral forelimb, twisted posture, empty jaw movements
**[37]**	Heiman et al.; 2014	6-OHDA MFB-lesioned hemi-parkinsonian mice	Reduced spontaneous use of forelimb contralateral to the lesion	Hyperkinetic movements of contralateral forelimb, twisted posture, empty jaw movements
**[38]**	Figge et al.; 2016	6-OHDA MFB-lesioned hemi-parkinsonian rats	Akinesia of the forelimb ipsilateral to the lesion	Hyperkinetic movements of contralateral forelimb, twisted posture, empty jaw movements

**[39]**	Figge et al.; 2017	6-OHDA MFB-lesioned hemi-parkinsonian rats	Akinesia of the forelimb ipsilateral to the lesion	Hyperkinetic movements of contralateral forelimb, twisted posture, empty jaw movements
**[41]**	Palermo et al.; 2020	PD patients	Resting tremor, rigidity, bradykinesia	Abnormal involuntary movements (twitches, jerks, twisting)
**[42]**	Jakobson et al.; 2013	PD patients	Resting tremor, rigidity, bradykinesia	Abnormal involuntary movements (twitches, jerks, twisting)
**[43]**	Jeong et al.; 2018	PD patients	Resting tremor, rigidity, bradykinesia	Abnormal involuntary movements (twitches, jerks, twisting)
**[45]**	Herz et al.; 2015	PD patients	Resting tremor, rigidity, bradykinesia	Abnormal involuntary movements (twitches, jerks, twisting)
**[46]**	Herz et al.; 2014	PD patients	Resting tremor, rigidity, bradykinesia	Abnormal involuntary movements (twitches, jerks, twisting)
**[47]**	Girasole et al.; 2018	6-OHDA Intrastriatally- or MFB-lesioned hemi-parkinsonian mice	Rotation ipsilateral to lesion	Hyperkinetic movements of contralateral forelimb, twisted posture, empty jaw movements
**[48]**	Ryan et al.; 2018	6-OHDA MFB-lesioned hemi-parkinsonian mice	Rotation ipsilateral to lesion	Hyperkinetic movements of contralateral forelimb, twisted posture, empty jaw movements
**[49]**	Parker et al.; 2018	6-OHDA MFB-lesioned hemi-parkinsonian mice	Rotation ipsilateral to lesion	Hyperkinetic movements of contralateral forelimb, twisted posture, empty jaw movements
**[50]**	Li et al.; 2013	Pitx3- parkinsonian mice	Bradykinesia in horizontal movements	Abnormal stereotypic paw movements, abnormal stereotypic vertical trunk movements
**[51]**	Singh et al; 2015	MPTP-lesioned parkinsonian non-human primates	Akinesia, bradykinesia, abnormal posture, absent checking behaviour	Abnormal flowing/flicking movements of limbs; sustained/transient sustained posture of leg, arm, trunk, or neck.
**[52]**	Belić et al.; 2016	6-OHDA MFB-lesioned hemi-parkinsonian rats	Assymetric posture, assymetric gait, reduced forelimb dexterity	Hyperkinetic movements of contralateral forelimb, twisted posture, empty jaw movements
**[53]**	Alberico et al.; 2017	6-OHDA MFB-lesioned hemi-parkinsonian mice	Rotation ipsilateral to lesion	Hyperkinetic movements of contralateral forelimb, twisted posture, empty jaw movements
**[54]**	Thiele et al.; 2014	6-OHDA MFB-lesioned hemi-parkinsonian mice	Rotation ipsilateral to lesion	Hyperkinetic movements of contralateral forelimb, twisted posture, empty jaw movements
**[55]**	Halje et al.; 2012	6-OHDA MFB-lesioned hemi-parkinsonian rats	Asymmetric posture, asymmetric gait, reduced forelimb dexterity	Hyperkinetic movements of contralateral forelimb, twisted posture, empty jaw movements
**[56]**	Perez et al.; 2017	6-OHDA MFB-lesioned hemi-parkinsonian mice	Asymmetry of forelimb movements	Hyperkinetic movements of contralateral forelimb, twisted posture, empty jaw movements
**[57]**	Shen et al.; 2020	6-OHDA MFB-lesioned hemi-parkinsonian mice	Asymmetry of forelimb movements	Hyperkinetic movements of contralateral forelimb, twisted posture, empty jaw movements
**[59]**	Loonen et al.; 2018	PD patients	Resting tremor, rigidity, bradykinesia	Abnormal involuntary movements (twitches, jerks, twisting)
**[60]**	Torkaman-Boutorabi et al.; 2012	PD patients	Resting tremor, rigidity, bradykinesia	Abnormal involuntary movements (twitches, jerks, twisting)
**[61]**	Sanitni et al.; 2012	6-OHDA MFB-lesioned hemi-parkinsonian mice	Akinesia contralateral to lesion	Hyperkinetic movements of contralateral forelimb, twisted posture, empty jaw movements
**[62]**	Calabrese et al.; 2020	6-OHDA MFB-lesioned hemi-parkinsonian rats	Contralateral rotations after apomorphine injection	Hyperkinetic movements of contralateral forelimb, twisted posture, empty jaw movements
**[63]**	Wu et al.; 2018	6-OHDA MFB-lesioned hemi-parkinsonian rats	Contralateral rotations after apomorphine injection	Hyperkinetic movements of contralateral forelimb, twisted posture, empty jaw movements

**[64]**	Jones-Tabah et al.; 2020	6-OHDA MFB-lesioned hemi-parkinsonian rats	Preferential use of forepaw ipsilateral to the lesion	Hyperkinetic movements of contralateral forelimb, twisted posture, empty jaw movements
**[65]**	Fieblinger et al.; 2014	6-OHDA MFB-lesioned hemi-parkinsonian rats and mice	Forepaw use asymmetry (mice), amphetamine-induced rotations (rats)	Hyperkinetic movements of contralateral forelimb, twisted posture, empty jaw movements
**[66]**	Alcacer et al.; 2012	6-OHDA MFB-lesioned hemi-parkinsonian mice	Forepaw use asymmetry	Hyperkinetic movements of contralateral forelimb, twisted posture, empty jaw movements
**[68]**	Södersten et al.; 2014	6-OHDA MFB-lesioned hemi-parkinsonian mice	Akinesia contralateral to lesion	Hyperkinetic movements of contralateral forelimb, twisted posture, empty jaw movements
**[69]**	Sellnow et al.; 2020	6-OHDA MFB-lesioned hemi-parkinsonian rats	Forepaw use asymmetry	Hyperkinetic movements of contralateral forelimb, twisted posture, empty jaw movements
**[70]**	Stanic et al.; 2016	6-OHDA MFB-lesioned hemi-parkinsonian rats	Contralateral rotations after apomorphine injection	Dystonic or choreiform torsion of the upper part of the body toward the side contralateral to the lesion; jerky and/or dystonic movements of the forelimb contralateral to the lesion; empty jaw movements and tongue protrusion
**[71]**	Divito et al.; 2015	6-OHDA MFB-lesioned hemi-parkinsonian mice	Asymmetry of forelimb movements	Dystonia, hyperkinesia, and/or stereotypies
**[73]**	Porras et al.; 2012	MPTP-lesioned parkinsonian non-human primates	Akinesia, bradykinesia, abnormal posture, absent checking behaviour	Choreic-athetoid (characterized by constant writhing and jerking motions), dystonic, and ballistic movements (large-amplitude flinging, flailing movements)
**[74]**	Berthet et al.; 2012	MPTP-lesioned parkinsonian non-human primates; 6-OHDA MFB-lesioned hemi-parkinsonian DAT-/Parkin-/UbG76V-GFP transgenic mice; 6-OHDA MFB-lesioned hemi-parkinsonian rats	Tremor, body posture (flexion of spine), vocalization, freezing arm movements, rigidity (primates); asymmetry of forelimb movements (mice and rats)	Abnormal flowing/flicking movements of limbs; sustained/transient sustained posture of leg, arm, trunk, or neck (primates); hyperkinetic movements of contralateral forelimb, twisted posture, empty jaw movements (mice and rats)
**[76]**	Zhang et al.; 2014	6-OHDA MFB-lesioned hemi-parkinsonian rats	Contralateral rotations after apomorphine injection	Hyperkinetic movements of contralateral forelimb, twisted posture, empty jaw movements
**[77]**	Albarrán-Bravo et al.; 2019	6-OHDA MFB-lesioned hemi-parkinsonian rats	Ipsilateral rotations	Hyperkinetic movements of contralateral forelimb, twisted posture, empty jaw movements
**[78]**	Solis et al.; 2017	6-OHDA MFB-lesioned hemi-parkinsonian mice	Asymmetry of forelimb movements	Hyperkinetic movements of contralateral forelimb, twisted posture, empty jaw movements
**[79]**	Payer et al.; 2016	PD patients	Resting tremor, rigidity, bradykinesia	Abnormal involuntary movements (twitches, jerks, twisting)
**[80]**	Castello et al.; 2020	6-OHDA MFB-lesioned hemi-parkinsonian mice	Asymmetry of forelimb movements	Hyperkinetic movements of contralateral forelimb, twisted posture, empty jaw movements
**[81]**	Bido et al.; 2015	6-OHDA MFB-lesioned hemi-parkinsonian Ras-GRF1/Ras-GRF2 knock-out mice	Asymmetry of forelimb movements	Hyperkinetic movements of contralateral forelimb, twisted posture, empty jaw movements
**[82]**	Fasano et al.; 2010	6-OHDA MFB-lesioned hemi-parkinsonian Ras-	Asymmetry of forelimb movements	Hyperkinetic movements of contralateral forelimb, twisted posture, empty jaw movements

		GRF1 knock-out mice		
**[83]**	Eshraghi et al.; 2020	6-OHDA MFB-lesioned hemi-parkinsonian RasGRP1 knock-out mice	Asymmetry of forelimb movements	Tight contralateral turns; contralateral dystonic posture of the neck and upper body toward the side contralateral to the lesion; jerky and fluttering movements of the limb contralateral to the side of the lesion
**[84]**	Cerovic et al.; 2015	6-OHDA MFB-lesioned hemi-parkinsonian Ras-GRF1 knock-out mice	Asymmetry of forelimb movements	Hyperkinetic movements of contralateral forelimb, twisted posture, empty jaw movements
**[85]**	Ruiz-DeDiego et al.; 2018	6-OHDA MFB-lesioned Nf1+/- or WT hemi-parkinsonian mice	Asymmetry of forelimb movements	Hyperkinetic movements of contralateral forelimb, twisted posture, empty jaw movements
**[86]**	Beck et al.; 2019	MPTP-lesioned parkinsonian non-human primates	Akinesia, bradykinesia, abnormal posture, absent checking behaviour	Abnormal flowing/flicking movements of limbs; sustained/transient sustained posture of leg, arm, trunk, or neck.
**[87]**	Cortés et al.; 2017	6-OHDA MFB-lesioned hemi-parkinsonian mice	Asymmetry of forelimb movements	Tight contralateral turns; contralateral dystonic posture of the neck and upper body; jerky, fluttering movements of the limb contralateral to the lesion; vacuous jaw movements, tongue protrusions
**[88]**	Bastide et al.; 2017	6-OHDA MFB-lesioned hemi-parkinsonian rats	Asymmetry of forelimb movements	Hyperkinetic movements of contralateral forelimb, twisted posture, empty jaw movements
**[89]**	Padovan-Neto et al.; 2013	6-OHDA MFB-lesioned hemi-parkinsonian rats	Contralateral rotations after apomorphine injection	Hyperkinetic movements of contralateral forelimb, twisted posture, empty jaw movements
**[91]**	Stanic et al.; 2017	6-OHDA MFB-lesioned hemi-parkinsonian rats	Contralateral rotations after apomorphine injection	Hyperkinetic movements of contralateral forelimb, twisted posture, empty jaw movements
**[92]**	Ba et al.; 2015	6-OHDA MFB-lesioned hemi-parkinsonian rats	Contralateral rotations after apomorphine injection	Hyperkinetic movements of contralateral forelimb, twisted posture, empty jaw movements
**[93]**	Lindenbach et al.; 2016	6-OHDA MFB-lesioned hemi-parkinsonian rats	Asymmetry of forelimb movements	Hyperkinetic movements of contralateral forelimb, twisted posture, empty jaw movements
**[94]**	Brumberg et al.; 2017	PD patients	Resting tremor, rigidity, bradykinesia	Abnormal involuntary movements (twitches, jerks, twisting)
**[95]**	Brugnoli et al.; 2020	6-OHDA MFB-lesioned hemi-parkinsonian rats	Asymmetry of forelimb movements	Hyperkinetic movements of contralateral forelimb, twisted posture, empty jaw movements
**[96]**	Shen et al.; 2018	MPTP-lesioned parkinsonian non-human primates; 6-OHDA MFB-lesioned hemi-parkinsonian mice	Tremor, body posture (flexion of spine), vocalization, freezing arm movements, rigidity (primates); asymmetry of forelimb movements (mice)	Abnormal flowing/flicking movements of limbs; sustained/transient sustained posture of leg, arm, trunk, or neck (primates); hyperkinetic movements of contralateral forelimb, twisted posture, empty jaw movements (mice)
**[97]**	Sahin et al.; 2014	6-OHDA MFB-lesioned hemi-parkinsonian rats	Ipsilateral rotations	Hyperkinetic movements of contralateral forelimb, twisted posture, empty jaw movements
**[98]**	Rylander et al.; 2010	MPTP-lesioned parkinsonian non-human primates; 6-OHDA MFB-lesioned hemi-parkinsonian mice	Tremor, body posture (flexion of spine), vocalization, freezing arm movements, rigidity (primates); asymmetry of forelimb movements (rats)	Abnormal flowing/flicking movements of limbs; sustained/transient sustained posture of leg, arm, trunk, or neck (primates); hyperkinetic movements of contralateral forelimb, twisted posture, empty jaw movements (rats)
**[99]**	Carta et al.; 2007	6-OHDA MFB-lesioned hemi-parkinsonian rats	Contralateral rotations after apomorphine injection	Hyperkinetic movements of contralateral forelimb, twisted posture, empty jaw movements

**[100]**	Tronci et al.; 2017	6-OHDA MFB-lesioned hemi-parkinsonian rats	Asymmetry of forelimb movements	Hyperkinetic movements of contralateral forelimb, twisted posture, empty jaw movements
**[101]**	Gagnon et al.; 2016	MPTP-lesioned parkinsonian non-human primates	Tremor, body posture (flexion of spine), vocalization, freezing arm movements, rigidity	Abnormal flowing/flicking movements of limbs; sustained/transient sustained posture of leg, arm, trunk, or neck.
**[102]**	Inden et al.; 2012	6-OHDA MFB-lesioned hemi-parkinsonian rats	Ipsilateral rotations	Hyperkinetic movements of contralateral forelimb, twisted posture, empty jaw movements
**[103]**	Bishop et al.; 2012	6-OHDA MFB-lesioned hemi-parkinsonian rats	Ipsilateral rotations	Hyperkinetic movements of contralateral forelimb, twisted posture, empty jaw movements
**[104]**	Miguelez et al.; 2016	6-OHDA MFB-lesioned hemi-parkinsonian rats	Ipsilateral rotations	Hyperkinetic movements of contralateral forelimb, twisted posture, empty jaw movements
**[105]**	Santos-Lobato et al.; 2020	PD patients	Resting tremor, rigidity, bradykinesia	Abnormal involuntary movements (twitches, jerks, twisting)
**[106]**	Hong et al.; 2020	PD patients	Resting tremor, rigidity, bradykinesia	Abnormal involuntary movements (twitches, jerks, twisting)
**[107]**	Lin et al.; 2019	PD patients	Resting tremor, rigidity, bradykinesia	Abnormal involuntary movements (twitches, jerks, twisting)
**[108]**	Poewe et al.; 2019	PD patients	Resting tremor, rigidity, bradykinesia	Abnormal involuntary movements (twitches, jerks, twisting)
**[109]**	Ramlackhansingh et al.; 2011	PD patients	Resting tremor, rigidity, bradykinesia	Abnormal involuntary movements (twitches, jerks, twisting)
**[110]**	Wills et al.; 2013	PD patients	Resting tremor, rigidity, bradykinesia	Abnormal involuntary movements (twitches, jerks, twisting)
**[111]**	Riggare et al.; 2017	PD patient	Resting tremor, rigidity, bradykinesia	Abnormal involuntary movements (twitches, jerks, twisting)
**[112]**	Popa et al.; 2020	PD patients	Resting tremor, rigidity, bradykinesia	Abnormal involuntary movements (twitches, jerks, twisting)
**[113]**	Wan et al.; 2017	6-OHDA MFB-lesioned hemi-parkinsonian rats	Ipsilateral rotations	Hyperkinetic movements of contralateral forelimb, twisted posture, empty jaw movements
**[114]**	Yang et al.; 2012	6-OHDA MFB-lesioned hemi-parkinsonian rats	Contralateral rotations after apomorphine injection	Hyperkinetic movements of contralateral forelimb, twisted posture, empty jaw movements
**[115]**	Xie et al.; 2014	6-OHDA MFB-lesioned hemi-parkinsonian rats	Contralateral rotations after apomorphine injection	Hyperkinetic movements of contralateral forelimb, twisted posture, empty jaw movements
**[116]**	Yang et al.; 2012	6-OHDA MFB-lesioned hemi-parkinsonian rats	Contralateral rotations after apomorphine injection	Hyperkinetic movements of contralateral forelimb, twisted posture, empty jaw movements
**[117]**	Cao et al.; 2016	6-OHDA MFB-lesioned hemi-parkinsonian rats	Contralateral rotations after apomorphine injection	Hyperkinetic movements of contralateral forelimb, twisted posture, empty jaw movements
**[118]**	Bido et al.; 2011	6-OHDA MFB-lesioned hemi-parkinsonian rats and mice	Contralateral rotations after amphetamine administration (rats); Forepaw use asymmetry (mice)	Hyperkinetic movements of contralateral forelimb, twisted posture, empty jaw movements
**[120]**	Hernandez et al.; 2019	PD patient	Resting tremor, rigidity, bradykinesia	Abnormal involuntary movements (twitches, jerks, twisting)
**[121]**	Sagarduy et al.; 2016	6-OHDA MFB-lesioned hemi-parkinsonian rats	Contralateral rotations after amphetamine administration	Hyperkinetic movements of contralateral forelimb, twisted posture, empty jaw movements
**[122]**	Vegas-Suárez et al.; 2020	6-OHDA MFB-lesioned hemi-parkinsonian rats	Ipsilateral rotations	Hyperkinetic movements of contralateral forelimb, twisted posture, empty jaw movements
**[123]**	Nahimi et al.; 2011	6-OHDA MFB-lesioned hemi-parkinsonian rats	Ipsilateral rotations	Turns contralateral to the lesioned side

**[124]**	Dupre et al.; 2016	6-OHDA MFB-lesioned hemi-parkinsonian rats	Asymmetry of forelimb movements	Dystonic posturing (twisting of the neck and torso directed toward the side of the body contralateral to the lesion); rapid, purposeless movements of the forelimb located on the side of the body contralateral to the lesion; repetitive openings and closings of the jaw; tongue protrusions
**[125]**	Lindenbach et al.; 2015	6-OHDA MFB-lesioned hemi-parkinsonian rats	Asymmetry of forelimb movements	Hyperkinetic movements of contralateral forelimb, twisted posture, empty jaw movements
**[126]**	Bhide et al.; 2013	6-OHDA MFB-lesioned hemi-parkinsonian rats	Contralateral rotations	Hyperkinetic movements of contralateral forelimb, twisted posture, empty jaw movements
**[127]**	Aboulghasemi et al.; 2018	6-OHDA MFB-lesioned hemi-parkinsonian rats	Contralateral rotations after apomorphine injection	Hyperkinetic movements of contralateral forelimb, twisted posture, empty jaw movements
**[128]**	Tronci et al.; 2013	6-OHDA MFB-lesioned hemi-parkinsonian female rats	Asymmetry of forelimb movements	Hyperkinetic movements of contralateral forelimb, twisted posture, empty jaw movements
**[130]**	Frau et al.; 2017	6-OHDA MFB-lesioned hemi-parkinsonian rats	Asymmetry of forelimb movements	Hyperkinetic movements of contralateral forelimb, twisted posture, empty jaw movements
**[134]**	Lindenbach et al.; 2011	6-OHDA MFB-lesioned hemi-parkinsonian rats	Asymmetry of forelimb movements	Hyperkinetic movements of contralateral forelimb, twisted posture, empty jaw movements
**[135]**	Bhide et al.; 2015	6-OHDA MFB-lesioned hemi-parkinsonian rats	Asymmetry of forelimb movements	Hyperkinetic movements of contralateral forelimb, twisted posture, empty jaw movements
**[136]**	Shi et al.; 2020	6-OHDA MFB-lesioned hemi-parkinsonian mice	Asymmetry of forelimb movements	Quick, repetitive, and uncontrolled limb movements; shaking and involuntary movements of the arms
**[139]**	Bordia et al.; 2013	6-OHDA MFB-lesioned hemi-parkinsonian rats	Asymmetry of forelimb movements	Hyperkinetic movements of contralateral forelimb, twisted posture, empty jaw movements
**[140]**	Leino et al.; 2018	6-OHDA MFB-lesioned hemi-parkinsonian female mice	Ipsilateral rotations	Hyperkinetic movements of contralateral forelimb, twisted posture, empty jaw movements
**[141]**	Teng et al.; 2014	6-OHDA MFB-lesioned hemi-parkinsonian rats	Contralateral rotations after apomorphine injection	Hyperkinetic movements of contralateral forelimb, twisted posture, empty jaw movements
**[142]**	Ogawa et al.; 2019	6-OHDA MFB-lesioned hemi-parkinsonian rats	Contralateral rotations after apomorphine injection	Hyperkinetic movements of contralateral forelimb, twisted posture, empty jaw movements
**[144]**	Cui et al.; 2014	6-OHDA MFB-lesioned hemi-parkinsonian rats	Asymmetry of forelimb movements	Hyperkinetic movements of contralateral forelimb, twisted posture, empty jaw movements
**[145]**	Shi et al.; 2015	6-OHDA MFB-lesioned hemi-parkinsonian rats	Contralateral rotations after apomorphine injection	Hyperkinetic movements of contralateral forelimb, twisted posture, empty jaw movements
**[146]**	Johansson et al.; 2001	6-OHDA MFB-lesioned hemi-parkinsonian rats	Contralateral rotations after amphetamine administration	Hyperkinetic movements of contralateral forelimb, twisted posture, empty jaw movements
**[147]**	Potts et al.; 2015	MPTP-lesioned parkinsonian non-human primates	Akinesia, bradykinesia, abnormal posture, absent checking behaviour	Abnormal flowing/flicking movements of limbs; sustained/transient sustained posture of leg, arm, trunk, or neck
**[148]**	Bartlett et al.; 2020	6-OHDA MFB-lesioned hemi-parkinsonian rats	Contralateral rotations after amphetamine administration	Hyperkinetic movements of contralateral forelimb, twisted posture, empty jaw movements
**[149]**	Bezard et al.; 2020	MPTP-lesioned parkinsonian non-human primates	Tremor, body posture (flexion of spine), vocalization, freezing of arm movements, rigidity	Choreic-athetoid (characterized by constant writhing and jerking motions), dystonic, and

				ballistic movements (large-amplitude flinging, flailing movements)
**[150]**	Arcuri et al.; 2018	6-OHDA MFB-lesioned hemi-parkinsonian rats	Contralateral rotations after amphetamine administration	Hyperkinetic movements of contralateral forelimb, twisted posture, empty jaw movements
**[152]**	Niccolini et al.; 2015	PD patients	Resting tremor, rigidity, bradykinesia	Abnormal involuntary movements (twitches, jerks, twisting)
**[153]**	Beck et al.; 2018	MPTP-lesioned parkinsonian non-human primates	Akinesia, bradykinesia, abnormal posture, absent checking behaviour	Abnormal flowing/flicking movements of limbs; sustained/transient sustained posture of leg, arm, trunk, or neck.
**[154]**	Sellnow et al.; 2019	6-OHDA MFB-lesioned hemi-parkinsonian rats	Asymmetry of forelimb movements	Hyperkinetic movements of contralateral forelimb, twisted posture, empty jaw movements
**[155]**	Marongiu et al.; 2016	6-OHDA MFB-lesioned hemi-parkinsonian mice	Asymmetry of forelimb movements	Hyperkinetic movements of contralateral forelimb, twisted posture, empty jaw movements
**[156]**	Breger et al.; 2017	6-OHDA MFB-lesioned hemi-parkinsonian rats	Asymmetry of forelimb movements; contralateral rotations after amphetamine administration	Hyperkinetic movements of contralateral forelimb, twisted posture, empty jaw movements
**[157]**	Ahmed et al.; 2015	6-OHDA MFB-lesioned hemi-parkinsonian rats	Contralateral rotations after apomorphine injection	Hyperkinetic movements of contralateral forelimb, twisted posture, empty jaw movements
**[159]**	Zhang et al.; 2019	6-OHDA MFB-lesioned hemi-parkinsonian rats	Contralateral rotations after apomorphine injection	Hyperkinetic movements of contralateral forelimb, twisted posture, empty jaw movements
**[160]**	Urs et al.; 2015	6-OHDA MFB-lesioned hemi-parkinsonian mice; MPTP-lesioned parkinsonian non-human primates	Asymmetry of forelimb movements (mice); akinesia, bradykinesia, abnormal posture, absent checking behaviour	Hyperkinetic movements of contralateral forelimb, twisted posture, empty jaw movements (mice); abnormal flowing/flicking movements of limbs; sustained/transient sustained posture of leg, arm, trunk, or neck.
**[161]**	Park et al.; 2014	6-OHDA MFB-lesioned hemi-parkinsonian mice	Asymmetry of forelimb movements	Hyperkinetic movements of contralateral forelimb, twisted posture, empty jaw movements
**[162]**	Leino et al.; 2018	6-OHDA MFB-lesioned hemi-parkinsonian mice	Contralateral rotations after amphetamine injection	Hyperkinetic movements of contralateral forelimb, twisted posture, empty jaw movements
**[163]**	Yang et al.; 2013	6-OHDA MFB-lesioned hemi-parkinsonian rats	Contralateral rotations after apomorphine injection	Hyperkinetic movements of contralateral forelimb, twisted posture, empty jaw movements
**[165]**	Steece-Collier et al.; 2019	6-OHDA MFB-lesioned hemi-parkinsonian rats	Asymmetry of forelimb movements	Dystonia, hyperkinesia, and/or stereotypies
**[166]**	Martinez et al.; 2015	6-OHDA MFB-lesioned hemi-parkinsonian rats	Contralateral rotations after amphetamine injection	Hyperkinetic movements of contralateral forelimb, twisted posture, empty jaw movements
**[167]**	Fernandez et al.; 2013	PD patients	Resting tremor, rigidity, bradykinesia	Abnormal involuntary movements (twitches, jerks, twisting)
**[168]**	Poewe et al.; 2016	PD patients	Resting tremor, rigidity, bradykinesia	Abnormal involuntary movements (twitches, jerks, twisting)
**[169]**	Băjenaru et al.; 2016	PD patients	Resting tremor, rigidity, bradykinesia	Abnormal involuntary movements (twitches, jerks, twisting)
**[170]**	Lopiano et al.; 2016	PD patients	Resting tremor, rigidity, bradykinesia	Abnormal involuntary movements (twitches, jerks, twisting)
**[171]**	Lopiano et al.; 2019	PD patients	Resting tremor, rigidity, bradykinesia	Abnormal involuntary movements (twitches, jerks, twisting)
**[172]**	Fernandez et al.; 2015	PD patients	Resting tremor, rigidity, bradykinesia	Abnormal involuntary movements (twitches, jerks, twisting)
**[173]**	Thakkar et al,; 2021	PD patients	Resting tremor, rigidity, bradykinesia	Abnormal involuntary movements (twitches, jerks, twisting)
**[174]**	Cruse et al.; 2018	PD patients	Resting tremor, rigidity, bradykinesia	Abnormal involuntary movements (twitches, jerks, twisting)

**[175]**	Olanow et al.; 2014	PD patients	Resting tremor, rigidity, bradykinesia	Abnormal involuntary movements (twitches, jerks, twisting)
**[176]**	Fabbri et al.; 2019	PD patients	Resting tremor, rigidity, bradykinesia	Abnormal involuntary movements (twitches, jerks, twisting)
**[177]**	Antonini et al.; 2015	PD patients	Resting tremor, rigidity, bradykinesia	Abnormal involuntary movements (twitches, jerks, twisting)
**[178]**	Morgante et al.; 2021	PD patients	Resting tremor, rigidity, bradykinesia	Abnormal involuntary movements (twitches, jerks, twisting)
**[179]**	Meloni et al.; 2017	PD patients	Resting tremor, rigidity, bradykinesia	Abnormal involuntary movements (twitches, jerks, twisting)
**[180]**	Santos Garcia et al.; 2016	PD patients	Resting tremor, rigidity, bradykinesia	Abnormal involuntary movements (twitches, jerks, twisting)
**[181]**	Garcia Ruiz et al.; 2008	PD patients	Resting tremor, rigidity, bradykinesia	Abnormal involuntary movements (twitches, jerks, twisting)
**[182]**	Houvenaghel et al.; 2018	PD patients	Resting tremor, rigidity, bradykinesia	Abnormal involuntary movements (twitches, jerks, twisting)
**[183]**	Katzenschlager et al.; 2021	PD patients	Resting tremor, rigidity, bradykinesia	Abnormal involuntary movements (twitches, jerks, twisting)
**[184]**	Henriksen et al.; 2021	PD patients	Resting tremor, rigidity, bradykinesia	Abnormal involuntary movements (twitches, jerks, twisting)
**[185]**	Papuć et al.; 2019	PD patients	Resting tremor, rigidity, bradykinesia	Abnormal involuntary movements (twitches, jerks, twisting)
**[186]**	Drapier et al.; 2016	PD patients	Resting tremor, rigidity, bradykinesia	Abnormal involuntary movements (twitches, jerks, twisting)
**[187]**	Olivola et al.; 2019	PD patients	Resting tremor, rigidity, bradykinesia	Abnormal involuntary movements (twitches, jerks, twisting)
**[188]**	Meira et al.; 2021	PD patients	Resting tremor, rigidity, bradykinesia	Abnormal involuntary movements (twitches, jerks, twisting)
**[189]**	LeWitt et al.; 2018	PD patients	Resting tremor, rigidity, bradykinesia	Abnormal involuntary movements (twitches, jerks, twisting)
**[190]**	Stocchi et al.; 2014	PD patients	Resting tremor, rigidity, bradykinesia	Abnormal involuntary movements (twitches, jerks, twisting)
**[191]**	Kong et al.; 2017	PD patients	Resting tremor, rigidity, bradykinesia	Abnormal involuntary movements (twitches, jerks, twisting)
**[192]**	Kim et al.; 2018	PD patients	Resting tremor, rigidity, bradykinesia	Abnormal involuntary movements (twitches, jerks, twisting)
**[193]**	Hauser et al.; 2021	PD patients	Resting tremor, rigidity, bradykinesia	Abnormal involuntary movements (twitches, jerks, twisting)
**[194]**	Pahwa et al.; 2015	PD patients	Resting tremor, rigidity, bradykinesia	Abnormal involuntary movements (twitches, jerks, twisting)
**[195]**	Oertel et al.; 2017	PD patients	Resting tremor, rigidity, bradykinesia	Abnormal involuntary movements (twitches, jerks, twisting)
**[196]**	Tanner et al.; 2020	PD patients	Resting tremor, rigidity, bradykinesia	Abnormal involuntary movements (twitches, jerks, twisting)
**[197]**	Hauser et al.; 2017	PD patients	Resting tremor, rigidity, bradykinesia	Abnormal involuntary movements (twitches, jerks, twisting)
**[198]**	Pahwa et al.; 2017	PD patients	Resting tremor, rigidity, bradykinesia	Abnormal involuntary movements (twitches, jerks, twisting)
**[201]**	Visanji et al.; 2009	MPTP-lesioned parkinsonian non-human primates	Akinesia, bradykinesia, abnormal posture, absent checking behaviour	Abnormal flowing/flicking movements of limbs; sustained/transient sustained posture of leg, arm, trunk, or neck
**[202]**	Utsumi et al.; 2013	PD patients	Resting tremor, rigidity, bradykinesia	Abnormal involuntary movements (twitches, jerks, twisting)
**[203]**	Svenningsson et al.; 2018	PD patients	Resting tremor, rigidity, bradykinesia	Abnormal involuntary movements (twitches, jerks, twisting)
**[204]**	Borgohain et al.; 2014	PD patients	Resting tremor, rigidity, bradykinesia	Abnormal involuntary movements (twitches, jerks, twisting)
**[205]**	Borgohain et al.; 2014	PD patients	Resting tremor, rigidity, bradykinesia	Abnormal involuntary movements (twitches, jerks, twisting)
**[206]**	Cattaneo et al.; 2020	PD patients	Resting tremor, rigidity, bradykinesia	Abnormal involuntary movements (twitches, jerks, twisting)

**[207]**	Gardoni et al.; 2018	6-OHDA MFB-lesioned hemi-parkinsonian rats	Contralateral rotations after apomorphine injection	Hyperkinetic movements of contralateral forelimb, twisted posture, empty jaw movements
**[208]**	Svenningsson et al.; 2015	PD patients	Resting tremor, rigidity, bradykinesia	Abnormal involuntary movements (twitches, jerks, twisting)
**[209]**	Meloni et al.; 2020	PD patients	Resting tremor, rigidity, bradykinesia	Abnormal involuntary movements (twitches, jerks, twisting)
**[210]**	Mestre et al.; 2014	PD patients	Resting tremor, rigidity, bradykinesia	Abnormal involuntary movements (twitches, jerks, twisting)
**[211]**	Fan et al.; 2020	PD patients	Resting tremor, rigidity, bradykinesia	Abnormal involuntary movements (twitches, jerks, twisting)
**[212]**	Liu et al.; 2019	PD patients	Resting tremor, rigidity, bradykinesia	Abnormal involuntary movements (twitches, jerks, twisting)
**[213]**	Ryu et al.; 2017	PD patients	Resting tremor, rigidity, bradykinesia	Abnormal involuntary movements (twitches, jerks, twisting)
**[214]**	Schuepbach et al.; 2013	PD patients	Resting tremor, rigidity, bradykinesia	Abnormal involuntary movements (twitches, jerks, twisting)
**[215]**	Weaver et al.; 2012	PD patients	Resting tremor, rigidity, bradykinesia	Abnormal involuntary movements (twitches, jerks, twisting)
**[216]**	Sobstyl et al.; 2017	PD patients	Resting tremor, rigidity, bradykinesia	Abnormal involuntary movements (twitches, jerks, twisting)
**[217]**	Kim et al.; 2015	PD patients	Resting tremor, rigidity, bradykinesia	Abnormal involuntary movements (twitches, jerks, twisting)
**[218]**	Lohse et al.; 2020	PD patients	Resting tremor, rigidity, bradykinesia	Abnormal involuntary movements (twitches, jerks, twisting)

Abbreviations: MFB, medial forebrain bundle; MPTP, methyl-4-phenyl-1,2,3,6-tetrahydropyridine; PD, Parkinson's disease; 6-hydroxydopamine, 6-OHDA.
